# Posicionamento – Protocolo de Reconexão dos Serviços de Cardiologia com os Pacientes Durante a Pandemia de COVID-19 – 2020

**DOI:** 10.36660/abc.20201004

**Published:** 2020-10-13

**Authors:** Marcio Sommer Bittencourt, Giuliano Generoso, Pedro Henrique M. Craveiro de Melo, Driele Peixoto, Érique José Farias Peixoto de Miranda, Evandro Tinoco Mesquita, Andréa Araujo Brandão, José Francisco Kerr Saraiva, Silvio Henrique Barberato, Fernando Bacal, Marcelo Antônio Cartaxo Queiroga Lopes

**Affiliations:** 1 Universidade de São Paulo Hospital Universitário São PauloSP Brasil Hospital Universitário da Universidade de São Paulo (USP), São Paulo, SP – Brasil; 2 Hospital Sírio Libanês São PauloSP Brasil Hospital Sírio Libanês, São Paulo, SP – Brasil; 3 Instituto do Câncer do Estado de São Paulo São PauloSP Brasil Instituto do Câncer do Estado de São Paulo (ICESP), São Paulo, SP – Brasil; 4 Bayer Pharmaceuticals SA São PauloSP Brasil Bayer Pharmaceuticals SA, São Paulo, SP – Brasil; 5 Universidade Federal Fluminense NiteróiRJ Brasil Universidade Federal Fluminense (UFF), Niterói, RJ – Brasil; 6 Centro de Ensino e Treinamento Edson de Godoy Bueno / UHG Rio de JaneiroRJ Brasil Centro de Ensino e Treinamento Edson de Godoy Bueno / UHG, Rio de Janeiro, RJ – Brasil; 7 Universidade do Estado do Rio de Janeiro Faculdade de Ciências Médicas Rio de JaneiroRJ Brasil Faculdade de Ciências Médicas da Universidade do Estado do Rio de Janeiro (FCM/UERJ), Rio de Janeiro, RJ – Brasil; 8 Sociedade Campineira de Educação e Instrução campinasSP Brasil Sociedade Campineira de Educação e Instrução, campinas, SP – Brasil; 9 CardioEco Centro de Diagnóstico Cardiovascular CuritibaPR Brasil CardioEco Centro de Diagnóstico Cardiovascular, Curitiba, PR – Brasil; 10 Universidade de São Paulo Faculdade de Medicina Instituto do Coração do Hospital das Clínicas São PauloSP Brasil Instituto do Coração do Hospital das Clínicas da Faculdade de Medicina da Universidade de São Paulo, São Paulo, SP – Brasil; 11 Hospital Israelita Albert Einstein São PauloSP Brasil Hospital Israelita Albert Einstein, São Paulo, SP – Brasil; 12 Hospital Alberto Urquiza Wanderley João PessoaPB Brasil Hospital Alberto Urquiza Wanderley, João Pessoa, PB – Brasil

**Realização:** Sociedade Brasileira de Cardiologia

**Conselho de Normatizações e Diretrizes (2020-2021):** Brivaldo Markman Filho, Antonio Carlos Sobral Sousa, Aurora Felice Castro Issa, Bruno Ramos Nascimento, Harry Correa Filho, Marcelo Luiz Campos Vieira

**Coordenador de Normatizações e Diretrizes (2020-2021):** Brivaldo Markman Filho

**Table:** 

O relatório abaixo lista as declarações de conflito de interesse conforme relatadas à SBC pelos especialistas durante o período de desenvolvimento desta diretriz, 2020.	
Especialista	Tipo de relacionamento com a indústria
Andréa Araujo Brandão	DECLARAÇÃO FINANCEIRA A - PAGAMENTO DE QUALQUER ESPÉCIE E DESDE QUE ECONOMICAMENTE APRECIÁVEIS, FEITOS A (i) VOCÊ, (ii) AO SEU CÔNJUGE/COMPANHEIRO OU A QUALQUER OUTRO MEMBRO QUE RESIDA COM VOCÊ, (iii) A QUALQUER PESSOA JURÍDICA EM QUE QUALQUER DESTES SEJA CONTROLADOR, SÓCIO, ACIONISTA OU PARTICIPANTE, DE FORMA DIRETA OU INDIRETA, RECEBIMENTO POR PALESTRAS, AULAS, ATUAÇÃO COMO PROCTOR DE TREINAMENTOS, REMUNERAÇÕES, HONORÁRIOS PAGOS POR PARTICIPAÇÕES EM CONSELHOS CONSULTIVOS, DE INVESTIGADORES, OU OUTROS COMITÊS, ETC. PROVENIENTES DA INDÚSTRIA FARMACÊUTICA, DE ÓRTESES, PRÓTESES, EQUIPAMENTOS E IMPLANTES, BRASILEIRAS OU ESTRANGEIRAS: - Servier: Palestras - Libbs: Hipertensão arterial - Novartis: Insuficiência cardíaca - EMS: Hipertensão arterial - Sandoz: Hipertensão arterial - Abbott: Hipertensão arterial OUTROS RELACIONAMENTOS: FINANCIAMENTO DE ATIVIDADES DE EDUCAÇÃO MÉDICA CONTINUADA, INCLUINDO VIAGENS, HOSPEDAGENS E INSCRIÇÕES PARA CONGRESSOS E CURSOS, PROVENIENTES DA INDÚSTRIA FARMACÊUTICA, DE ÓRTESES, PRÓTESES, EQUIPAMENTOS E IMPLANTES, BRASILEIRAS OU ESTRANGEIRAS: - Servier: Hipertensão arterial
Driele Peixoto	Nada a ser declarado
Érique José Farias Peixoto de Miranda,	DECLARAÇÃO FINANCEIRA A - PAGAMENTO DE QUALQUER ESPÉCIE E DESDE QUE ECONOMICAMENTE APRECIÁVEIS, FEITOS A (i) VOCÊ, (ii) AO SEU CÔNJUGE/COMPANHEIRO OU A QUALQUER OUTRO MEMBRO QUE RESIDA COM VOCÊ, (iii) A QUALQUER PESSOA JURÍDICA EM QUE QUALQUER DESTES SEJA CONTROLADOR, SÓCIO, ACIONISTA OU PARTICIPANTE, DE FORMA DIRETA OU INDIRETA, RECEBIMENTO POR PALESTRAS, AULAS, ATUAÇÃO COMO PROCTOR DE TREINAMENTOS, REMUNERAÇÕES, HONORÁRIOS PAGOS POR PARTICIPAÇÕES EM CONSELHOS CONSULTIVOS, DE INVESTIGADORES, OU OUTROS COMITÊS, ETC. PROVENIENTES DA INDÚSTRIA FARMACÊUTICA, DE ÓRTESES, PRÓTESES, EQUIPAMENTOS E IMPLANTES, BRASILEIRAS OU ESTRANGEIRAS:w - Bayer: Xarelto OUTROS RELACIONAMENTOS VÍNCULO EMPREGATÍCIO COM A INDÚSTRIA FARMACÊUTICA, DE ÓRTESES, PRÓTESES, EQUIPAMENTOS E IMPLANTES, BRASILEIRAS OU ESTRANGEIRAS, ASSIM COMO SE TEM RELAÇÃO VÍNCULO EMPREGATÍCIO COM OPERADORAS DE PLANOS DE SAÚDE OU EM AUDITORIAS MÉDICAS (INCLUINDO MEIO PERÍODO) DURANTE O ANO PARA O QUAL VOCÊ ESTÁ DECLARANDO: - Bayer
Evandro Tinoco Mesquita	OUTROS RELACIONAMENTOS VÍNCULO EMPREGATÍCIO COM A INDÚSTRIA FARMACÊUTICA, DE ÓRTESES, PRÓTESES, EQUIPAMENTOS E IMPLANTES, BRASILEIRAS OU ESTRANGEIRAS, ASSIM COMO SE TEM RELAÇÃO VÍNCULO EMPREGATÍCIO COM OPERADORAS DE PLANOS DE SAÚDE OU EM AUDITORIAS MÉDICAS (INCLUINDO MEIO PERÍODO) DURANTE O ANO PARA O QUAL VOCÊ ESTÁ DECLARANDO: - UnitedHealth Group
Fernando Bacal	DECLARAÇÃO FINANCEIRA A - PAGAMENTO DE QUALQUER ESPÉCIE E DESDE QUE ECONOMICAMENTE APRECIÁVEIS, FEITOS A (i) VOCÊ, (ii) AO SEU CÔNJUGE/COMPANHEIRO OU A QUALQUER OUTRO MEMBRO QUE RESIDA COM VOCÊ, (iii) A QUALQUER PESSOA JURÍDICA EM QUE QUALQUER DESTES SEJA CONTROLADOR, SÓCIO, ACIONISTA OU PARTICIPANTE, DE FORMA DIRETA OU INDIRETA, RECEBIMENTO POR PALESTRAS, AULAS, ATUAÇÃO COMO PROCTOR DE TREINAMENTOS, REMUNERAÇÕES, HONORÁRIOS PAGOS POR PARTICIPAÇÕES EM CONSELHOS CONSULTIVOS, DE INVESTIGADORES, OU OUTROS COMITÊS, ETC. PROVENIENTES DA INDÚSTRIA FARMACÊUTICA, DE ÓRTESES, PRÓTESES, EQUIPAMENTOS E IMPLANTES, BRASILEIRAS OU ESTRANGEIRAS - Novartis: Entresto
Giuliano Generoso	Nada a ser declarado
José Francisco Kerr Saraiva	Nada a ser declarado
Marcelo Antônio Cartaxo Queiroga Lopes	Nada a ser declarado
Marcio Sommer Bittencourt	DECLARAÇÃO FINANCEIRA A - PAGAMENTO DE QUALQUER ESPÉCIE E DESDE QUE ECONOMICAMENTE APRECIÁVEIS, FEITOS A (i) VOCÊ, (ii) AO SEU CÔNJUGE/COMPANHEIRO OU A QUALQUER OUTRO MEMBRO QUE RESIDA COM VOCÊ, (iii) A QUALQUER PESSOA JURÍDICA EM QUE QUALQUER DESTES SEJA CONTROLADOR, SÓCIO, ACIONISTA OU PARTICIPANTE, DE FORMA DIRETA OU INDIRETA, RECEBIMENTO POR PALESTRAS, AULAS, ATUAÇÃO COMO PROCTOR DE TREINAMENTOS, REMUNERAÇÕES, HONORÁRIOS PAGOS POR PARTICIPAÇÕES EM CONSELHOS CONSULTIVOS, DE INVESTIGADORES, OU OUTROS COMITÊS, ETC. PROVENIENTES DA INDÚSTRIA FARMACÊUTICA, DE ÓRTESES, PRÓTESES, EQUIPAMENTOS E IMPLANTES, BRASILEIRAS OU ESTRANGEIRAS: - Sanofi: Pesquisa - EMS: Palestras Novo Nordisk: Palestras C - FINANCIAMENTO DE PESQUISA (PESSOAL), CUJAS RECEITAS TENHAM SIDO PROVENIENTES DA INDÚSTRIA FARMACÊUTICA, DE ÓRTESES, PRÓTESES, EQUIPAMENTOS E IMPLANTES, BRASILEIRAS OU ESTRANGEIRAS: - Sanofi: Pesquisa
Pedro Henrique M. Craveiro de Melo	Nada a ser declarado
Silvio Henrique Barberato	Nada a ser declarado

## 1. Introdução e Conceitos Gerais

A pandemia resultante da infecção pelo novo coronavírus (SARS-CoV-2), denominada COVID-19, teve início em Wuhan, na China, em dezembro de 2019.^1^^,^^2^ Já acometeu milhões de pessoas em todo o mundo, resultando em centenas de milhares de mortes.

A transmissão do vírus ocorre principalmente de pessoa a pessoa^1^^,^^2^ de forma direta pelas vias respiratórias ou pelo contato indireto com superfícies e objetos contaminados. As infecções respiratórias acontecem através da transmissão de gotículas contendo vírus (> 5 *μ*m com extensão aproximada de 1,5 m) ou aerossóis (≤ 5 *μ*m com extensão aproximada de 8 m) exalados por indivíduos infectados.^3^^,^^4^ Dados recentes sugerem possível transmissão pelo ar. O contato de gotículas respiratórias contaminadas, eliminadas pela respiração, fala, espirro e tosse de pessoas infectadas, com as mucosas de olhos, boca e nariz de indivíduos suscetíveis resulta na transmissão do SARS-CoV-2. Com a expansão da pandemia de COVID-19 e o rápido aumento de casos, estudos demonstraram a viabilidade do vírus no ambiente e o papel das superfícies contaminadas na transmissão hospitalar da COVID-19.^5^ Com base na transmissão por gotículas, os Centros para Controle e Prevenção de Doenças dos Estados Unidos recomendam distanciamento físico de no mínimo 1,5 m e higienização das mãos para redução da propagação do vírus.

A apresentação clínica da COVID-19 pode variar desde uma forma leve a moderada, que é mais frequente e acomete 80% dos casos, até uma mais grave, que acomete os outros 20% com quadro comparável ao que no Brasil se define como síndrome respiratória aguda grave (SRAG).^6^ Os sintomas mais sensíveis da forma leve/moderada são tosse não produtiva e febre, que pode ou não ocorrer no momento da apresentação, ao passo que os mais específicos são alteração do olfato e do paladar.^7^^,^^8^ A SRAG é definida como uma síndrome gripal (SG) associada a dispneia/desconforto respiratório ou pressão persistente no tórax ou saturação de O_2_ menor que 95% em ar ambiente ou coloração azulada dos lábios ou rosto, com taquipneia, cianose e grave acometimento pulmonar.

Devido ao potencial de disseminação do vírus, à apresentação clínica grave em uma parte dos casos e à inexistência de tratamento viral específico, ou mesmo de vacina, medidas não farmacológicas são as intervenções mais eficazes até o presente momento.^9^^,^^10^ Essas intervenções não farmacológicas têm como objetivo reduzir a transmissão interpessoal do vírus por diminuição do contato entre infectados e suscetíveis, quer seja por aumento da distância entre as pessoas, redução da intensidade e da duração do contato, quer seja pelo uso de medidas e dispositivos físicos ou químicos que impeçam a passagem do vírus de uma pessoa para outra.

As intervenções não farmacológicas podem ser divididas em:

- Distanciamento físico: consiste no afastamento físico de no mínimo 2,0 m entre os indivíduos, tendo como exemplo o fechamento de escolas e de espaços de alta concentração de pessoas e restrição de viagens.
- Medidas de bloqueio de transmissão: higienização das mãos com água e sabão ou álcool, etiqueta da tosse e utilização de máscaras ou outras barreiras físicas de contato.
- Identificação e isolamento de casos suspeitos ou confirmados: manter os casos em isolamento individual.
- Quarentena de contatos: rastreamento ativo dos contatos do caso e instituição de isolamento social, além de observação da evolução clínica para casos suspeitos.

Infelizmente, a população sofre as repercussões dessa crise humanitária não apenas pela COVID-19, como também pelos danos colaterais associados a atrasos e redução no atendimento de outras doenças em ambiente de pronto-socorro, redução do acesso a cuidados de doenças crônicas em ambiente ambulatorial e maior exposição aos fatores de risco para o desenvolvimento de doenças (sedentarismo, obesidade, ansiedade e estresse emocional). Somem-se a isso perdas econômicas, impacto psicológico e *burnout* associado à pandemia. São duas as principais circunstâncias em que ocorre essa diminuição no cuidado adequado à saúde. Primeiro, há redução de consultas médicas e exames de rotina. Essa disrupção no atendimento de pacientes crônicos pode levar a descompensação aguda de condições como hipertensão arterial, diabetes e insuficiência cardíaca. Da mesma forma, podem ser enfrentadas consequências graves relacionadas à redução da procura e à demora no atendimento hospitalar de urgência e emergência em circunstâncias como síndromes coronarianas agudas, insuficiência cardíaca aguda e acidentes vasculares encefálicos.^11^^-^^13^

Buscando minimizar essas adversidades, a Sociedade Brasileira de Cardiologia (SBC) compilou orientações baseadas em evidências recentes, mesmo que limitadas e por vezes fundamentadas em opiniões de especialistas ou relatos preliminares, para criar um direcionamento estruturado visando à reconexão do médico com os pacientes de forma planejada na reabertura de serviços de cardiologia. Tal direcionamento tem por objetivo diminuir o risco tanto para pacientes e acompanhantes quanto para profissionais de saúde envolvidos nas atividades clínicas da Cardiologia.

As recomendações contidas neste documento são baseadas nas evidências disponíveis no momento da sua elaboração e na opinião de especialistas. O conhecimento em relação à COVID-19 evolui de forma dinâmica e rápida, logo, os protocolos para reintrodução com segurança de atendimentos médicos, procedimentos invasivos e procedimentos não invasivos estão em constante evolução e adaptação. Este projeto foi idealizado pela SBC como uma fonte de referência para seus associados. As recomendações apresentadas, contudo, não devem ser usadas como única base para a definição de protocolos locais, devendo outras fontes atualizadas ser consideradas à medida que o conhecimento na área evolui.

O presente posicionamento busca alinhar as seguintes demandas:

- Minimizar o risco de transmissão do SARS-CoV-2 entre pacientes, profissionais de saúde e outros envolvidos no atendimento.
- Identificar precocemente casos suspeitos de COVID-19 e implementar procedimento de triagem para atribuir níveis adequados de atendimento, reduzir o risco de complicações da COVID-19 e das doenças cardiovasculares presentes, assim como diminuir o risco de transmissão.
- Fornecer informações sobre cuidados relacionados à COVID-19 de maneira segura e confiável para pacientes e profissionais de saúde.
- Reduzir os impactos negativos nos pronto-atendimentos e internações hospitalares pela falta do tratamento ambulatorial de condições pré-existentes.
- Otimizar a utilização de equipamento de proteção individual (EPI).

Assim, para promover atendimento de excelência, mantendo a segurança do profissional de saúde e do paciente com doença cardiovascular, devemos admitir as seguintes premissas:

- Como a apresentação clínica é variável, a definição da presença ou ausência de infecção por SARS-CoV-2 pode não ser possível apenas com a avaliação clínica inicial. Protocolos claros de triagem devem ser utilizados para minimizar o risco de pacientes suspeitos circularem no ambiente de saúde, salvo para atendimento de urgência ou emergência. Caso necessária, essa circulação deve acontecer com o menor risco de contaminação possível.
- Todos os profissionais de saúde devem ser continuadamente treinados com relação a boas práticas, protocolos institucionais e fluxogramas de atendimento.
- Os atendimentos de emergência devem seguir o mesmo protocolo dos cuidados com pacientes com COVID-19 confirmada/suspeita, pois não é possível descartar COVID-19 em tempo hábil para o atendimento adequado, que deve continuar disponível em sua capacidade total.
- Os procedimentos eletivos e semi-eletivos serão retomados após planejamento, em menor escala do que sua capacidade prévia, com reavaliação contínua de comitê diretor responsável, sempre respeitando as autorizações e eventuais restrições à circulação, com abertura de serviços médicos definida pelas autoridades competentes.
- As rotas de acesso aos equipamentos diagnósticos devem ser destacadas para os serviços de transporte e indicadas para pacientes e demais funcionários que não pertençam ao setor médico de atendimento com o objetivo de minimizar o contato entre profissionais da saúde e pacientes e a sua exposição. Para pacientes com suspeita ou confirmação de COVID-19 em que o exame ou procedimento seja necessário, os fluxos e as rotas de atendimento, bem como as áreas de espera correspondentes, devem ser separados das vias normais do paciente sem suspeita de infecção por SARS-CoV-2.
- Redução do fluxo de pessoal e da exposição da equipe (profissionais administrativos da recepção, profissionais de higiene, profissionais de saúde).

Finalmente, para hierarquização dos fluxos de atendimento nas diversas áreas da assistência médica em cardiologia, é necessário definir o perfil clínico do paciente, a proximidade física do paciente com a equipe de saúde e o tipo de contato durante o atendimento, além do nível de urgência para o tratamento da doença cardíaca.

O momento da reintrodução do atendimento em cardiologia deve estar alinhado com as políticas institucionais e seguir as recomendações das autoridades competentes. Considerações importantes incluem a incidência local de pacientes com infecção por SARS-CoV-2 e a tendência do número de casos, bem como recursos institucionais disponíveis, incluindo instalações, recursos humanos e cadeias de suprimento de EPI. Por exemplo, pode ser necessária a suspensão temporária de atendimentos eletivos se o estoque de EPI estiver limitado, a fim de evitar o desabastecimento do atendimento de urgência e emergência.

À medida que as regiões do país caminhem para situação de controle da epidemia de COVID-19 após o impacto transformador imposto aos serviços de saúde, tais orientações poderão sofrer flexibilização regionalizada. Além disso, o acompanhamento de casos de pacientes e prestadores de serviço é crucial para a identificação da progressão de possível transmissão local e necessidade de aumento do nível de resposta dentro de instituições médicas, particularmente as que possuem leitos de internação. Portanto, é essencial que a comunidade médica permaneça vigilante e atenta ao caráter dinâmico das recomendações tanto na esfera estadual ou municipal como até mesmo dentro de seu consultório, clínica ou instituição hospitalar.

## 2. Perfis do Atendimento, Clínico do Paciente e do Ambiente de Atendimento

### a. Orientações Gerais Aplicáveis a Todos os Ambientes de Atendimento

Para obter êxito na retomada das atividades, é essencial que todo serviço de atendimento médico defina um plano local elaborado com participação ativa dos membros da equipe médica, dos profissionais com experiência no controle de infecções e segurança do paciente e de demais profissionais de saúde envolvidos na prática assistencial. Assim, destacamos um plano de medidas iniciais aplicável a todos os níveis de atendimento.

**Figura 1:**
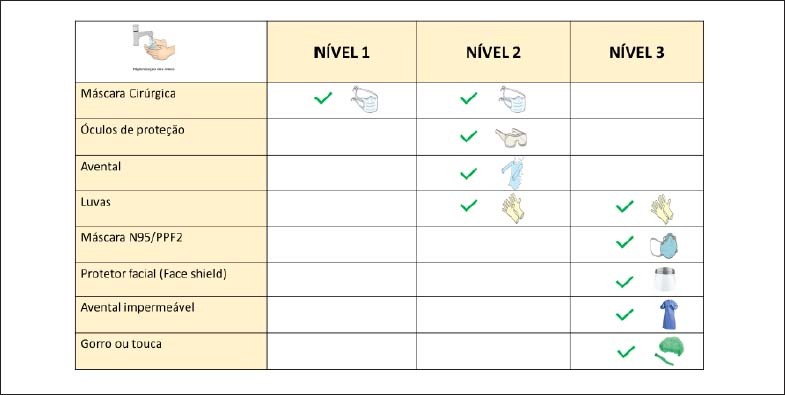
Orientações de EPI de acordo com os níveis de risco. Para o Nível 2, os óculos de proteção podem ser substituídos por protetor facial. No Nível 3, o protetor facial é obrigatório e não pode ser substituído por óculos de proteção. Fonte adaptada: GVIMS/GGTES/ANVISA.

- Iniciar o retorno às atividades com capacidade reduzida, por exemplo algo próximo de 25% da capacidade máxima pré-pandemia, permitindo a implementação controlada das intervenções e a avaliação do fluxo de atendimento. Após constatação de que o fluxo ocorre de forma adequada, pode-se realizar o aumento escalonado no volume de atendimentos. No entanto, não há expectativa de que o fluxo atinja volumes pré-pandemia durante o período de flexibilização em razão das adaptações necessárias. Deve-se considerar esse aspecto dentro do potencial impacto na sustentabilidade financeira do serviço de saúde.
- Devem-se priorizar os pacientes mais sintomáticos e com doenças de maior probabilidade de descompensação nas próximas semanas ou meses, reduzindo a chance de internações e de complicações a curto e médio prazo e auxiliando no combate à sobrecarga do sistema de internação hospitalar.
- Adequar estrutura física, com o objetivo de garantir distanciamento físico, como demarcações sinalizadas no chão, associadas à utilização de barreiras físicas no ambiente, como painéis de acrílico ou vidro.
- Garantir que os suprimentos de higiene e limpeza, tais como álcool gel, lenços de papel e sabonete para as mãos, estejam prontamente disponíveis e facilmente acessíveis, além de assegurar o descarte adequado de resíduos.
- Elaborar lembretes visuais, como cartazes, placas e *posters*, que devem ser disponibilizados para os pacientes tanto por via digital quanto afixados na entrada do serviço e em locais estratégicos com informações principais sobre higienização das mãos, etiqueta da tosse e principais sinais e sintomas da COVID-19.
- Manter toda a equipe com uso contínuo de EPI adequado durante todo o tempo de atendimento (Figura 1).
- Certificar-se de que todos os pacientes e acompanhantes com mais de 2 anos estejam com o rosto coberto com máscara e, para aqueles que não estejam, oferecer máscaras faciais no momento da triagem. É importante ressaltar que alguns locais de atendimento de saúde com características próprias, como hospitais, podem ter regulamentação específica que torne obrigatório o uso de máscaras cirúrgicas descartáveis. Essas especificações devem ser consideradas pelos serviços de assistência na definição de qual máscara exigir do paciente durante o atendimento.
- Para serviços médicos de maior porte com equipe de funcionários maior, deve-se definir um comitê de retomada para discussão continuada de ajustes nas intervenções e no volume de atendimento a fim de reduzir o risco de transmissão.
- No dia anterior à consulta, aplicar o Questionário de Sintomas e Exposição (Suplemento A) por meio eletrônico ou contato telefônico. Todo paciente deve preencher o questionário de sintomas por telefone (verbal) ou por via eletrônica ou impressa.
- Aferir a temperatura corporal de todos os pacientes na chegada para o atendimento.
- Caso seja necessária a realização de consulta ou exame em paciente com suspeita ou confirmação de COVID-19, deve-se reservar uma sala separada para o atendimento.
- No ambiente hospitalar, gerenciar mecanismos de barreira para os pacientes ambulatoriais, criando entradas e saídas “limpas”, ou seja, sem contato com os pacientes internados.
- Nos atendimentos de urgência e emergência (definição na seção 2b), onde não é possível realizar triagem prévia nem adiar o atendimento, aplicar de forma objetiva, após avaliação cardiológica padrão, o Questionário de Sintomas e Exposição para definir o *status* epidemiológico.

Em caso de qualquer resposta positiva ao questionário (Suplemento A):

- Assegurar que o paciente esteja usando máscara cirúrgica.
- Manter o paciente em uma sala de espera separada, com portas fechadas e, se possível, sinalizada.
- Manter distanciamento físico de 2,0 m.
- O supervisor responsável e o prestador do tratamento médico devem ser comunicados e o atendimento deve ser realizado com a utilização de EPI adequado por todos os membros da equipe.
- Serviços que ofereçam atendimento de urgência e emergência devem definir fluxo de atendimento específico de acordo com a estrutura local disponível.

### b. Definição do Perfil de Atendimento Cardiológico Quanto à sua Urgência

Para definição do protocolo de atendimento, é necessário considerar a condição clínica e a urgência do paciente atendido. Definimos aqui quatros perfis de atendimento de acordo com as características clínicas do paciente e risco de piora por adiamento do cuidado:

- Perfil A: Situação de atendimento de emergência: quando o atendimento, a avaliação e a intervenção devem ser realizados nos próximos **minutos ou horas**.
- Perfil B: Situação de atendimento de urgência: quando o atendimento, a avaliação e a intervenção devem ser realizados nos próximos **dias**.
- Perfil C: Situação de atendimento semi-eletivo: quando o atendimento, a avaliação e a intervenção devem ser realizados nas próximas **semanas,** idealmente antes de 3 meses.
- Perfil D: Situação de atendimento eletivo: quando não há necessidade de atendimento, avaliação e intervenção a curto prazo, podendo ser postergados para além de 3 meses.

A Tabela 1 detalha as principais apresentações de doenças cardiovasculares de acordo com a classificação acima.

**Tabela 1:** Principais apresentações clínicas de doenças cardiovasculares de acordo com a situação de urgência do atendimento

**Situações de atendimento de emergência**
Infarto do miocárdio com elevação do segmento ST
Síndrome coronariana aguda de alto risco
Arritmia ventricular refratária
FA por síndrome de pré-excitação ventricular
Síndromes aórticas agudas
Correções de disfunções anatômicas cardíacas levando a choque cardiogênico
Disfunção em DACM intra- ou extracorpóreo
Bradiarritmias e taquiarritmias com repercussão hemodinâmica
Tamponamento cardíaco
Edema agudo de pulmão
**Situações de atendimento de urgência**
Síndrome coronariana aguda de moderado risco
Síndrome coronariana aguda de baixo risco
Disfunção valvar anatomicamente importante, sintomática
FA e taquicardia supraventricular recorrente com repercussão clínica
Insuficiência cardíaca descompensada classe funcional NYHA IV
Transplante cardíaco de urgência
Tumores cardíacos de alto risco
Cardiopatia congênita grave, sintomática
Hipertrigliceridemia grave > 1.000 mg/l
Disfunção de qualquer componente de DCEI
DCEI com bateria em fim de vida
**Situações de atendimento semi-eletivo**
Angina estável
Hipertensão não controlada
Insuficiência cardíaca descompensada classe funcional NYHA III
Diabetes não controlado
Disfunção valvar anatomicamente moderada
Aneurisma de aorta
Disfunção valvar anatomicamente importante, assintomática
Pacientes em avaliação para implante de DACM
Nova alteração anatômica cardíaca em paciente previamente hígido
DCEI com bateria apresentando indicação de substituição não urgente
**Situações de atendimento eletivo**
Demais casos

DACM: dispositivo de assistência circulatória mecânica; DCEI: dispositivo cardíaco eletrônico implantável; FA: fibrilação atrial; NYHA: New York Heart Association.

### c. Definição do Perfil Clínico do Paciente (*Status* para COVID-19)

A apresentação clínica compatível com COVID-19 suspeita ou confirmada deve considerar a definição de SG e de SRAG conforme detalhado na Tabela 2.

**Tabela 2:** Definição de síndrome gripal e síndrome respiratória aguda grave

Classificação	Características clínicas
Síndrome gripal	Sintomas respiratórios, como tosse, coriza, dor de garganta, com ou sem febre*
Síndrome Respiratória Aguda Grave	Saturação de O_2_ < 95% em ar ambiente E/OU frequência respiratória ≥ 24 ipm

* Febre pode não estar presente em alguns casos, como idosos e imunodeprimidos. Nessas situações, a avaliação clínica deve ser levada em consideração.

O critério clínico, entretanto, não permite estabelecer a etiologia, devendo outros agentes entrar no diagnóstico diferencial, conforme evidências epidemiológicas, dos exames laboratoriais e dos achados radiológicos.

Com relação à infecção por SARS-CoV-2, o perfil clínico de um paciente pode ser classificado como:

- *Com suspeita de COVID-19*: caso suspeito de SG ou SRAG por critério clínico, radiológico ou laboratorial presuntivo.
- *Com confirmação de COVID-19 ativa*: caso suspeito de SG ou SRAG com infecção pelo SARS-CoV-2 confirmada por método laboratorial definitivo (RT-PCR para SARS-CoV-2 detectado) ou sorologia IgM E:
  ◦ Menos de 10 dias da data de início dos sintomas ou da data do exame, se assintomático; OU
  ◦ Menos de 3 dias do último sintoma relacionado à COVID-19.
- *Com confirmação de COVID-19 curada*: COVID-19 confirmada com evidências de melhora do quadro clínico, definida por ausência de febre por > 3 dias e melhora dos sintomas respiratórios (tosse, falta de ar) E:
  ◦ Pelo menos de 10 dias decorridos desde os primeiros sintomas; OU
  ◦ Sorologia IgG reagente com história clínica compatível com COVID-19 prévia.
- *Contato de caso suspeito ou confirmado*: aquele que esteve em contato (no trabalho, domicílio) há menos de 14 dias com indivíduo classificado em um dos três *status* acima.
- *Assintomático sem contato recente com caso*: aquele que não apresenta nenhum sintoma sugestivo de infecção por SARS-CoV-2 nos últimos 10 dias nem contato com caso suspeito ou confirmado nos últimos 14 dias.
- *Caso descartado***:** Caso suspeito de SG ou SRAG sem confirmação da infecção pelo SARS-CoV-2 por método laboratorial definitivo (RT-PCR para SARS-CoV-2 detectado) durante a janela de oportunidade de diagnóstico **OU** confirmação laboratorial para outro agente etiológico, como vírus influenza ou vírus sincicial respiratório. Em casos de alta suspeição, pode ser necessário repetir o teste de RT-PCR em 48 horas em razão de sua sensibilidade limitada.

### d. Definição do Ambiente de Atendimento

O perfil clínico descrito acima é útil não somente para definir a urgência da realização do atendimento médico, mas também seu modelo estrutural de abordagem. Sempre que possível, o risco de exposição da equipe de saúde e do paciente e seus acompanhantes deve ser minimizado, priorizando-se o atendimento mais seguro, porém efetivo. Para isso, o ambiente de atendimento foi classificado de acordo com a distância e o tempo de contato entre o paciente e a equipe de saúde:

- *Ambiente I: Atendimento remoto,* onde não há qualquer contato físico entre o paciente e a equipe.
- *Ambiente II: Ambiente de contato moderado,* onde há contato presencial entre paciente e equipe, com curto tempo de exposição (< 15 minutos) e/ou maior distanciamento físico (> 1,5 m).
- *Ambiente III: Ambiente de contato próximo,* há contato físico entre paciente e equipe próximo ou prolongado (> 15 minutos, em ambiente fechado).
- *Ambiente IV: Contato invasivo e manipulação potencial de vias aéreas,* há contato direto com o paciente ou manipulação de vias aéreas do paciente e exposição a aerossóis.

Para tornar mais objetivo, exemplos de diversos perfis de ambiente para atendimentos cardiológicos de rotina encontram-se detalhados na Tabela 3. É importante enfatizar o estímulo à emissão de laudos/diagnósticos, sempre que possível, via acesso remoto.

**Tabela 3:** Classificação dos ambientes de atendimento cardiológico de acordo com o contato interpessoal

**Ambientes de trabalho da equipe de cardiologia**
**Atendimento remoto**
Sistemas de suporte à decisão
Teleconsultoria, incluindo orientação telefônica
Telerregulação
Telediagnóstico e laudos à distância
Teleducação
**Ambiente de contato moderado**
Estacionamento (serviço de manobrista)
Profissionais da recepção e equipe administrativo em geral
Profissionais de segurança
**Ambiente de contato próximo**
Consultas médicas presenciais
Equipe presencial em exames de medicina nuclear
Equipe presencial em exames de imagem (tomografia computadorizada, ressonância magnética, ecocardiograma)
Equipe presencial em métodos gráficos em cardiologia
**Contato invasivo e manipulação potencial de vias áreas**
Oroscopia em exame físico, teste de esforço
Ecocardiograma transesofágico
Procedimentos invasivos em eletrofisiologia
Procedimentos invasivos em cardiologia intervencionista

## 3. Medidas de Precaução e Isolamento COVID-19

**Figura 2:**
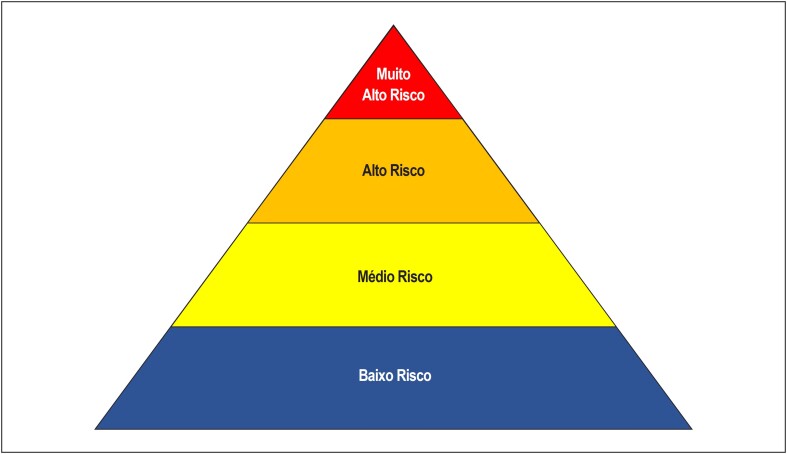
Pirâmide de risco ocupacional para COVID-19 (adaptada de Occupational Safety and Health Administration - OSHA).^14^

O risco ocupacional de exposição ao SARS-CoV-2 pode ser dividido em quatro níveis: muito alto, alto, médio e baixo risco (Figura 2). A avaliação do risco ocupacional depende da distância e do tempo de contato entre o profissional e o paciente, do tipo de procedimento realizado e do seu risco de geração de aerossóis, além do perfil clínico do paciente com relação à infecção pelo SARS-CoV-2.

Dessa forma, além das precauções-padrão, as seguintes devem ser implementadas pelos serviços de saúde:

◦ Precauções contra contato.
◦ Precauções contra gotículas.
◦ Precauções contra aerossóis.

Alguns procedimentos que podem gerar aerossóis são: entubação ou aspiração traqueal, ventilação mecânica não invasiva, ressuscitação cardiopulmonar, ventilação manual antes da entubação, coleta de amostras nasotraqueais, broncoscopia. Para os atendimentos em que esses procedimentos são rotineiros ou potencialmente necessários, as precauções contra gotículas devem ser substituídas pelas precauções contra aerossóis.

Portanto, os EPI necessários para o atendimento de casos suspeitos ou confirmados consistem em:

### a. Medidas de Precaução-Padrão

Instituídas para todos os pacientes e englobam:

- Higienização das mãos.
- Uso de máscara – durante a vigência da epidemia conforme detalhado abaixo para o nível 1 de EPI.

Se houver risco de exposição a sangue ou secreções:

- Uso de luvas de procedimento.
- Uso de óculos de proteção e avental.

Compreendem também o descarte adequado.

### b. Medidas de Precaução contra Contato + Gotículas

- Máscara cirúrgica.
- Óculos de proteção ou protetor facial (*face shield*).
- Avental com gramatura mínima de 30 g/m^2^.
- Luvas de procedimentos.

### c. Medidas de Precaução contra Contato + Aerossóis

- Máscara N95/PPF2 ou equivalente.
- Protetor facial (*face shield*).
- Avental impermeável.
- Gorro.
- Luvas de procedimentos.

Além dos procedimentos geradores de aerossóis, quando o paciente estiver com expectoração abundante, sangramento, vômitos ou diarreia, o profissional de saúde deve usar avental impermeável. O uso racional do EPI nos serviços de saúde é necessário e, por ser o EPI considerado um recurso finito, pode haver alteração nas recomendações de sua utilização em razão de planos de contingência durante a pandemia.

### d. Recomendação de EPI de Acordo com a Complexidade dos Procedimentos e o Perfil Clínico do Paciente Figuras 1 e 2, Tabela 4)

**Tabela 4:** Definição do nível de EPI a ser utilizado de acordo com o risco apresentado

Risco	Tipo de exposição	Tipo de atendimento	Nível de EPI
Muito alto risco	Exposição a pacientes com confirmação ou suspeita de COVID-19	Oroscopia e oftalmoscopia ETE, teste de esforço Procedimentos invasivos em eletrofisiologia e em cardiologia intervencionista	Nível 3
Contato invasivo de vias aéreas	Procedimentos que geram aerossóis		
Alto risco	Exposição a pacientes com confirmação ou suspeita de COVID-19	Exames de medicina nuclear Exames de imagem (TC, RM, ETT) Métodos gráficos em cardiologia	Nível 2
Ambiente de contato próximo			
Médio risco	Contato frequente e próximo	Profissionais da recepção *Staff* administrativo em geral Estacionamento Consultas presenciais	Nível 1
Ambiente de contato moderado			
Baixo risco	Sem contato com pacientes	Telerregulação Telediagnóstico Teleducação	Sem EPI específico
Atendimento remoto			

ETE: ecocardiograma transesofágico; ETT: ecocardiograma transtorácico; RM: ressonância magnética; TC: tomografia computadorizada. Para pacientes com suspeita ou confirmação de COVID-19, as consultas presenciais devem ser realizadas com EPI nível 2. Algumas diretrizes consideram que exames de imagem e métodos gráficos, exceto teste ergométrico, podem ser realizados somente com máscara cirúrgica, particularmente quando há baixa probabilidade de COVID-19 ativa, como quando o caso é de paciente com COVID-19 curada ou em casos sem sintomas ou contato recente e exame negativo para COVID-19 recente.

**Nível 1 de EPI:** Para médio risco, ambiente de contato moderado.

**Medidas de Precaução-Padrão**

Indicadas para procedimentos NÃO invasivos quando realizados em caráter eletivo E em pacientes sem suspeita de COVID-19.

- Profissionais responsáveis por atividades que não envolvam contato a menos de 2,0 m com os pacientes poderão utilizar máscara de tecido.
- Se não for garantido o distanciamento de 2,0 m do paciente, deve-se utilizar máscara cirúrgica durante as atividades (Tabela 5).
- Instituir barreiras físicas, de forma a favorecer o distanciamento maior que 2,0 m, por exemplo, placas de acrílico e faixas no piso (Tabela 5).

**Tabela 5:** Precauções-padrão de acordo com o distanciamento

Tipo de precaução	Padrão
Profissionais > 2,0 m do paciente	Máscara de tecido
Profissionais < 2,0 m do paciente	Máscara cirúrgica

A máscara de tecido não deve ser utilizada pela equipe de saúde no atendimento.

**Nível 2 de EPI**: Para alto risco, ambiente de contato próximo.

**Medidas de Precaução contra Contato + Gotículas**

Indicadas para procedimentos NÃO invasivos em caráter de emergência OU em pacientes com confirmação OU suspeita de infecção por SARS-CoV-2.

**Nível 3 de EPI**: Para muito alto risco, contato invasivo de vias aéreas.

**Medidas de Precaução contra Contato + Aerossóis**

Indicadas para procedimentos invasivos e potencialmente **geradores de aerossóis** quando realizados:

- em caráter eletivo E em pacientes sem suspeita de COVID-19.
- em caráter de emergência OU em pacientes com confirmação OU suspeita de infecção por SARS-CoV-2.

Nota: Em procedimentos invasivos, substituir os EPI (luvas, aventais) por equipamento devidamente esterilizado.

Importante destacar que a máscara de tecido não é um EPI, por isso não deve ser usada por profissionais de saúde ou de apoio em situações em que haja indicação de uso de máscara cirúrgica (durante a assistência ou contato direto, a menos de 2,0 m de pacientes) ou de máscara N95/PFF2 (durante a realização de procedimentos potencialmente geradores de aerossóis).

### e. Limpeza e Desinfecção de Superfícies

Não há recomendação diferenciada para a limpeza e a desinfecção de superfícies após o contato com casos suspeitos ou confirmados de COVID-19. Porém, é fundamental que os serviços revisem os procedimentos operacionais de limpeza e desinfecção de ambientes e superfícies para garantir melhores práticas e aumento da periodicidade de limpeza.^14^^,^^15^^,^^16^

- Com relação aos equipamentos específicos no manejo cardiológico, todos deverão ser limpos a cada término de jornada de trabalho, ainda com os profissionais usando EPI e evitando contato com os materiais infectados.
- Monitores e superfícies de máquina de ultrassom poderão ser cobertos com filme plástico para diminuir risco de contaminação e facilitar a limpeza.
- Após a saída do paciente com suspeita ou confirmação, realizar a limpeza/desinfecção, com saneante de uso habitual, dos equipamentos e de todas as áreas que entraram em contato com o paciente, além das áreas tocadas pelos profissionais de saúde durante o atendimento.

## 4. Estratégia de Abordagem no Contexto do Ambiente de Atendimento

### a. Ambiente de Atendimento Presencial

**Table:** 

**Classificação: Ambiente II (contato moderado)**
**Risco: Baixo a moderado**

Os ambientes de atendimento presencial são aqueles onde os serviços de saúde são prestados no âmbito extra-hospitalar. Incluem unidades básicas de saúde, ambulatórios médicos de especialidades, ambulatórios hospitalares, clínicas e consultórios médicos. São parte essencial na resposta do sistema de saúde, já que há um potencial aumento de desfechos cardiovasculares a médio prazo em razão do déficit na assistência médica a essa população. As adaptações do protocolo de atendimento devem incluir as seguintes modificações na rotina:^16^

#### Agendamento

- A marcação de consultas deve ser realizada prioritariamente por meios não presenciais (telefônico ou on-line), em plataformas dedicadas ou via aplicativos de mensagens.
- Os agendamentos devem iniciar com horários espaçados e intervalos entre os atendimentos, permitindo que um paciente deixe o ambiente, incluindo a sala de espera, antes da chegada do próximo atendimento, minimizando o contato entre pacientes.
- A triagem de pacientes com sintomas respiratórios deve ser realizada no agendamento e na confirmação da consulta, utilizando questionário (Suplemento A).
- Deve-se orientar e exigir o uso de máscara pelo paciente e acompanhante independentemente da presença de sintomas ou das respostas ao questionário.
- Deve-se orientar quanto ao comparecimento ao atendimento sem acompanhante, sempre que possível. Quando estritamente necessário, indicar a aceitação de apenas um acompanhante, que também deve responder ao mesmo questionário de sintomas.

#### Estrutura Física/ Sala de Espera

- Manter os ambientes ventilados (ar condicionado com exaustão, que garanta as trocas de ar, ou manter as janelas abertas).
- Reduzir o número de pessoas da equipe da recepção que terá contato físico com o paciente.
- Utilizar barreiras físicas na recepção para reduzir contato, como placas de acrílico e demarcações no chão para distanciamento físico de no mínimo 2,0 m.
- Adicionar alertas visuais nas entradas e em locais estratégicos com instruções sobre higiene das mãos, etiqueta da tosse e sinais e sintomas suspeitos para COVID-19.
- Disponibilizar lenço descartável para higiene nasal ou etiqueta da tosse na sala de espera. Prover lixeira com acionamento por pedal para o descarte de lenços de papel.
- Disponibilizar dispensadores com preparações alcoólicas para a higiene das mãos e lavatório/pia com dispensador de sabonete líquido, suporte para papel toalha, papel toalha, lixeira com tampa e abertura sem contato manual.
- Estimular espera em ambiente aberto ou externo, respeitando o distanciamento físico de 2,0 m.
- Sinalizar distância mínima entre os assentos de 2,0 m.
- Utilizar copos descartáveis para água e café.
- Retirar objetos compartilhados, como revistas e jornais.
- Orientar higienização das mãos antes e após o preenchimento de fichas, o uso de canetas e o pagamento com cartão. Higienizar a máquina de cartão e, para facilitar o processo, a máquina poderá ser envolvida em plástico filme, sempre procedendo à higienização após cada uso.
- Fornecer máscara cirúrgica para pacientes com sintomas respiratórios e orientar o uso correto durante toda a sua permanência na unidade, caso o atendimento seja estritamente necessário. Pacientes com atendimentos eletivos ou semi-eletivos devem ser orientados a retornar ao seu domicílio para isolamento individual e investigação de COVID-19, com reagendamento para atendimento posterior. As máscaras devem ser trocadas sempre que estiverem sujas ou úmidas.
- Caso o paciente apresente sintomas respiratórios, encaminhá-lo para uma sala de isolamento ou área separada, isolada dos demais pacientes.
- Aumentar a frequência de limpeza e desinfecção do ambiente, objetos e superfícies mais tocados.
- Não há recomendação do uso de tapetes com soluções saneantes na entrada do serviço ou cabines de desinfecção, pois não têm eficácia documentada.
- Nenhum tipo de produto deve ser borrifado nos pacientes, acompanhantes ou equipe assistencial, pelo alto risco de intoxicação e falta de evidência científica para uso.

#### Acompanhante

Recomendar que os pacientes compareçam sem acompanhantes.

- Caso identificada a necessidade de acompanhante para paciente com autocuidado limitado ou por outra razão, permitir presença de apenas uma pessoa não pertencente ao grupo de risco (Tabela 6).
- No dia anterior à consulta, aplicar o Questionário de Sintomas e Exposição também ao acompanhante.
- Não se recomenda a realização de consultas de pacientes que necessitem de atendimento eletivo ou semi-eletivo e que tenham acompanhantes sintomáticos ou com contato recente com casos suspeitos ou confirmados de COVID-19.
- Os acompanhantes devem seguir todas as orientações e recomendações fornecidas aos pacientes.

**Tabela 6:** Avaliação de grupo de risco para triagem dos acompanhantes

Idade > 65 anos
Obesidade
Hipertensão arterial sistêmica
Diabetes mellitus
Tabagismo
Insuficiência cardíaca
Doença pulmonar obstrutiva crônica
Doença renal crônica
Anemia falciforme
Estados de imunossupressão contínua (transplantes, infecção pelo HIV, doença oncológica, uso crônico de imunossupressores)
Asma (moderada a grave)
Doença cerebrovascular
Gravidez
Doença hepática

### b. Atendimento Via Telemedicina

**Table:** 

**Classificação: Ambiente I (remoto)**
**Risco: Nenhum**

O atendimento via telemedicina é peça fundamental no retorno racional às atividades e foi pormenorizado na *Diretriz da Sociedade Brasileira de Cardiologia sobre Telemedicina na Cardiologia - 2019*.^17^ No contexto dessa diretriz, devemos enfatizar o conceito de teleconsulta e telemonitoramento e destacar que seu uso é recomendado, exceto em casos de urgência e emergência, onde o atendimento presencial é necessário. Além da recomendação geral para seu uso, vale salientar que o atendimento à distância deve ser preferido também quando o prestador de serviços de saúde é do grupo de risco.

- Todas as consultas médicas por telemedicina devem obrigatoriamente ocorrer via teleatendimento com sincronia de áudio e vídeo, além de equipamentos que garantam o sigilo médico do atendimento e o registro adequado da consulta em prontuário.
- A telemedicina pode ser utilizada rotineiramente para a realização de pré-consulta pelo médico, que deve avaliar a necessidade de o paciente comparecer presencialmente, definir o risco do quadro clínico, encaminhar para hospital ou mesmo resolver o caso com atendimento completo por telemedicina.
- O atendimento remoto deve ser feito preferencialmente com ferramentas de certificação digital e assinatura eletrônica, configurando originalidade das informações, bem como segurança para o médico e o paciente.

### c. Acompanhamento Remoto

**Table:** 

**Classificação: Ambiente I (remoto)**
**Risco: Nenhum**

O telemonitoramento de sinais vitais necessários e de resultados de exames pode ocorrer por outros meios de acesso remoto. São exemplos de telemonitoramento: seguimento de sintomas na insuficiência cardíaca, níveis pressóricos, telemetria de dispositivo cardíaco eletrônico implantável. Encaixam-se no atendimento de telemonitoramento o controle de exames laboratoriais à distância, como função renal, controle de anticoagulação etc.

O telemonitoramento pode e deve ser utilizado para acompanhamento remoto de pacientes com o objetivo de diminuir o número de consultas presenciais, reduzindo o trânsito e a mobilidade dos pacientes, sempre que possível.

## 5. Estratégia de Abordagem no Contexto dos Exames Não Invasivos

### a. Métodos Gráficos em Cardiologia

Os exames complementares que compreendem os métodos gráficos, de acordo com a proximidade entre paciente e profissional, podem ser classificados com relação ao ambiente da seguinte forma:

*Atendimento Remoto/ à Distância*

- Monitorização Residencial da Pressão Arterial (MRPA).

*Ambiente de Contato Moderado*

- Eletrocardiografia de repouso.
- Holter.
- Monitorização Ambulatorial da Pressão Arterial (MAPA).

*Ambiente de Contato Próximo*

- Teste da inclinação (*Tilt-table test*).

Assim como orientado para as demais áreas da cardiologia, deve-se realizar o agendamento, por vias não presenciais, em horários programados para reduzir o número de pessoas ao mesmo tempo na sala de espera. O Questionário de Sintomas e Exposição deve ser aplicado no dia anterior ao procedimento, tanto para o paciente quanto para o acompanhante. Esse último deve estar presente apenas se imprescindível ao atendimento e não deve pertencer ao grupo de risco para infecção por SARS-CoV-2 (Tabela 6).

No contexto específico dos métodos gráficos, devem-se estimular os meios não presenciais de diagnóstico, como o acesso remoto e o telediagnóstico em tempo real, ferramentas que reduzem tanto o número de profissionais de saúde expostos como o seu tempo de contato com o paciente. Em casos em que esses exames não podem ser postergados, a orientação para a reabertura inclui:

- Emitir laudo por meio não presencial de eletrocardiograma, Holter, MAPA, MRPA.
- Dar prioridade ao uso da MRPA no lugar do MAPA, quando possível.
- Seguir os protocolos institucionais de contato e higienização/limpeza para instalação e devolução dos aparelhos de Holter e MAPA.
- Considerar outro método alternativo ao teste ergométrico para avaliar isquemia miocárdica, com associação de técnicas de imagem ao estresse farmacológico para reduzir a exposição da equipe a gotículas e aerossóis.

### b. Teste de Esforço

**Table:** 

**Classificação: Contato invasivo de vias aéreas**
**Risco: Muito alto risco**

É ferramenta importante, visto sua ampla utilização, sendo o principal método de avaliação de isquemia em diversos serviços no país. No entanto, deve-se ter cautela durante o período de transmissão comunitária sustentada, pois há maior risco de transmissão pelo aumento da frequência respiratória durante o teste, aumento da emissão de gotículas, ambiente fechado e longo tempo de permanência em sala. Portanto, exames eletivos e semi-eletivos devem ser avaliados caso a caso. Recomenda-se considerar o adiamento desse teste durante a fase de maior transmissão comunitária do SARS-CoV-2.

Ainda, nos casos raros em que o exame possa ser considerado necessário em pacientes com suspeita ou confirmação de infecção por SARS-CoV-2 recente, realizá-lo observando-se as precauções específicas, Nível 3 (precaução contato + aerossóis). Devido ao fato de as evidências científicas ainda serem incertas com relação a esse tipo de exposição, há a possibilidade de mudança na recomendação em futuras publicações.

Com relação ao teste de inclinação, embora haja exposição prolongada do profissional de saúde, não há aumento de trabalho respiratório, podendo-se inferir que o risco de transmissão não é tão aumentando. Assim, orienta-se precaução Nível 2.

- No caso de realização de teste ergométrico, o paciente deve permanecer com máscara cirúrgica durante toda a duração do exame.^18^
- Não se recomenda a presença de outras pessoas (familiares, pais, treinadores etc.) na sala de ergometria.
- Devem-se priorizar manguitos automáticos de pressão arterial sempre que disponíveis.
- As equipes de saúde devem utilizar os EPI preconizados conforme a proximidade com o paciente, precaução específica de nível 3.
- As equipes de saúde devem realizar o exame mantendo o maior distanciamento físico possível do paciente, que deve ser, no mínimo, de 2,0 m.
- Recomenda-se que a sala de ergometria seja ventilada ativamente, além de intervalo de tempo entre os testes ergométricos individuais idealmente de pelo menos 60 minutos em cada esteira para permitir tempo suficiente para a adequada higienização dos equipamentos.
- Todo o equipamento utilizado para a realização do exame deve ser adequadamente higienizado entre cada paciente avaliado.

### c. Ecocardiograma

A reabertura dos serviços de ecocardiograma deve contemplar as medidas já descritas em relação ao agendamento não presencial, ao maior intervalo entre os agendamentos, às recomendações para acompanhantes, à limpeza e higienização do ambiente e ao distanciamento físico. Em virtude da grande proximidade entre o operador responsável pelo exame e o paciente durante a aquisição das imagens, deve-se considerar o adiamento de exames eletivos e semi-eletivos durante o período de transmissão comunitária sustentada, particularmente para pacientes de grupos de risco (Tabela 7).

**Tabela 7:** Classificação de prioridade para realização do ecocardiograma

**Situação de atendimento de urgência e emergência**
Sintomas cardiovasculares recentes clinicamente relevantes (insuficiência cardíaca CF III ou IV, síncope de provável origem cardíaca, dor torácica, arritmias)
Procedimento recente que requer acompanhamento urgente
Arritmias pós-implante de dispositivo
Derrame pericárdico
Avaliação no pós-operatório de cirurgia cardíaca
Avaliação inicial antes do início do tratamento medicamentoso oncológico (quimioterapia/imunoterapia)
Suspeita de endocardite infecciosa com alta probabilidade pré-teste
**Situação de atendimento semi-eletivo**
Paciente assintomático, porém com doença cardíaca crônica que requer monitoramento para progressão
Avaliação de doença valvar estável (estenose ou insuficiência das valvas aórtica e mitral)
Hipertensão pulmonar
Progressão da doença após intervenção (coarctação recorrente, estenose de condutos)
Terapia não cardiológica que exige monitoramento contínuo
Estimativa da pressão sistólica da artéria pulmonar em pacientes recebendo terapia específica
Avaliação de rejeição após transplante cardíaco
Tratamento para a doença de Kawasaki
Avaliação de acompanhamento da função do DAV em pacientes estáveis
Ecocardiograma pré-operatório, não urgente
**Situação de atendimento eletivo**
Acompanhamento de rotina para doenças crônicas: hipertensão, doença arterial coronariana, avaliação anual da doença da aorta ou função protética da válvula (função normal no exame prévio e sem novos sintomas)

CF: classe funcional; DAV: dispositivo de assistência ventricular.

### d. Ecocardiograma Transtorácico (ETT)

**Table:** 

**Classificação: Ambiente de contato próximo**
**Risco: Alto Risco**

- Na fase inicial da reabertura, agendar primeiro os exames de alta prioridade e, na sequência, os de média prioridade, conforme momento epidemiológico local e sucesso da reabertura.
- Aplicar com antecedência os questionários de sintomas e exposição e repetir a triagem antes dos procedimentos.
- Utilização de EPI adequado pela equipe.

### e. Ecocardiograma Transesofágico (ETE)

**Table:** 

**Classificação: Contato invasivo de vias aéreas**
**Risco: Muito Alto Risco**

As considerações gerais para realização de ETE seguem os mesmos princípios descritos acima. Entretanto, são recomendadas precauções adicionais em virtude do potencial de dispersão de aerossóis relacionada a estímulo do reflexo da tosse em pacientes com via aérea não protegida, recomendando-se a utilização adequada de EPI completo e universal e higienização meticulosa da sala de exames e dos equipamentos.

Idealmente, os procedimentos que podem gerar aerossóis devem ser realizados em uma unidade de isolamento respiratório com pressão negativa e filtro HEPA (*High Efficiency Particulate Arrestance*). Na ausência desse tipo de unidade, deve-se colocar o paciente em um quarto individual com portas fechadas e restringir o número de profissionais durante esses procedimentos. Em razão do alto risco que o ETE representa, sua indicação deve ser avaliada caso a caso durante a pandemia.

#### EPI Recomendados

Utilização de precaução específica de nível 3 (precaução contra contato + aerossóis) para todos os operadores em contato com a via aérea do paciente e a equipe de apoio que estiver na sala.

#### Higienização do Equipamento

O detalhamento da higienização do equipamento de ETE está além do escopo do presente documento. Outros documentos de protocolos de higienização e desinfecção fornecidos pelos serviços de controle de infecção hospitalar e normativas técnicas institucionais devem ser seguidos de forma rotineira.

### f. Ecocardiograma de Estresse

**Table:** 

**Classificação: Ambiente de contato próximo**
**Risco: Alto Risco**

O ecocardiograma realizado com estresse físico promove as mesmas alterações da frequência respiratória descritas para o teste ergométrico, com o agravante de que o operador não pode se distanciar adequadamente do paciente. Por esses motivos, o estresse físico deve ser considerado procedimento de exceção e não ser realizado rotineiramente até o controle adequado da transmissão comunitária da COVID-19. Considera-se alternativa adequada a realização do ecocardiograma de estresse com agentes farmacológicos ou a realização de outros métodos de imagem quando necessário. Em caso de exceção em que o ecocardiograma com estresse físico tenha que ser realizado, recomenda-se a utilização de precaução específica de nível 3 (precaução contra contato + aerossóis) por todos os profissionais presentes no ambiente.

O ecocardiograma de estresse com dobutamina ou outros agentes farmacológicos deve ser a alternativa preferencial durante o período vigente, devendo-se seguir todas as precauções recomendadas para a realização do ecocardiograma de repouso.

#### Pacientes com COVID-19 Confirmada ou Suspeita

Os exames de ecocardiograma em pacientes com quadro agudo e diagnóstico de COVID-19 confirmado ou suspeito devem ser realizados apenas em casos de urgência e emergência, nos quais se espera que o resultado do exame tenha real impacto na conduta clínica. Recomenda-se preferencialmente o uso de aparelhos portáteis com atenção especial à proteção da equipe.

- Planejar com antecedência o ecocardiograma para análise apenas das janelas necessárias para a tomada de decisão.
- Utilizar o tempo ao lado do paciente apenas para adquirir as imagens e vídeos, realizando-se posteriormente as medidas em *software* dedicado.
- Remanejar os profissionais em formação e ecocardiografistas menos experientes para as áreas não COVID-19 no intuito de minimizar o tempo de realização do exame.
- As equipes de saúde devem utilizar os EPI preconizados conforme a proximidade com o paciente, precaução específica de nível 3 (precaução contato + aerossóis).

### g. Tomografia Computadorizada (TC) e Ressonância Magnética (RM) do Coração

**Table:** 

**Classificação: Ambiente de contato moderado**
**Risco: Médio Risco**

Em todo o mundo, os departamentos de radiologia e suas salas de modalidade de imagem não foram projetadas com medidas restritivas de transmissão de doenças infecciosas. Entretanto, os exames de imagem são ferramentas essenciais no diagnóstico e tratamento da COVID-19 e suas complicações.

São obrigatórias as medidas iniciais já descritas para outros exames relacionadas ao agendamento não presencial, ao maior intervalo entre os atendimentos da agenda, às recomendações para acompanhantes, à limpeza/higienização do ambiente e ao distanciamento físico.

No processo de adaptação para o funcionamento sob as atuais condições incomuns, os pacientes com suspeita ou confirmação de COVID-19 devem ser submetidos preferencialmente aos exames em equipamentos dedicados a esse perfil clínico, a fim de evitar a contaminação cruzada entre populações de pacientes infectados e não infectados. Esse aspecto é de particular importância, pois a realização de TC para a investigação de pacientes com suspeita ou quadro confirmado de COVID-19 é frequente. Caso isso não seja possível, é necessário definir, no equipamento disponível, intervalos de tempo dedicados ao perfil clínico “COVID-19 suspeita ou confirmada”, de preferência no final do dia. Devido à viabilidade do SARS-CoV-2 em vários tipos de superfícies e condições ambientais, é obrigatória a limpeza da instalação de imagem após cada paciente com COVID-19 suspeita ou confirmada antes da obtenção de imagens de um paciente sem suspeita ou confirmação de COVID-19, devendo a limpeza ser realizada de acordo com os protocolos institucionais.

Deve-se considerar o reagendamento de exames eletivos e semi-eletivos durante o período de transmissão comunitária mais intensa da COVID-19. Exames de urgência e emergência devem ser considerados conforme necessidade clínica e expectativa de definição de conduta de tratamento baseada no resultado dos exames.

Nos casos de pacientes internados, a imagem cardiovascular pode ser usada para substituir exames invasivos ou com manipulação de vias aéreas, como o ETE e a cineangiocoronariografia. Assim, em casos selecionados, pode-se lançar mão da TC cardíaca para pesquisa de trombo em apêndice atrial esquerdo ou angiotomografia de coronárias em síndrome coronariana aguda sem elevação do segmento ST. Para pacientes com perfil clínico A ou B, em que o exame seja considerado necessário e não substituível, ou em casos selecionados de pacientes com perfil clínico C em que o exame seja necessário, os protocolos de cuidado de nível 2 devem ser recomendados. Devido ao baixo grau de evidência, esta recomendação é feita por segurança e outras diretrizes podem recomendar somente o uso de máscara cirúrgica em pacientes sem suspeita de COVID-19. Esta estratégia é particularmente aceitável em casos de COVID-19 curada ou em casos sem sintomas e exame negativo recente.

A RM cardíaca, pelo seu tempo prolongado de execução e, consequentemente, maior exposição da equipe, deve ser indicada de forma seletiva em casos suspeitos ou confirmados de COVID-19, como no diagnóstico diferencial entre miocardite, síndrome de Takotsubo e infarto do miocárdio sem lesões coronarianas obstrutivas (MINOCA). As indicações e os níveis de prioridade para TC e RM estão detalhados na Tabela 8.

**Tabela 8:** Indicações de tomografia computadorizada / ressonância magnética de coração durante a pandemia por COVID-19

**Situação de atendimento de urgência/alternativa**	
Síndrome coronariana aguda sem elevação do segmento ST – diagnóstico diferencial, descartar:	
	Doença arterial coronariana
	Miocardite aguda
	MINOCA
	Síndrome de Takotsubo
Descartar embolia pulmonar (em protocolos de triplo descarte)	
Detecção de trombo em átrio esquerdo na FA em paciente hospitalizado	
Disfunção valvar com descompensação aguda	
Suspeita de endocardite valvar	
Planejamento pré-TAVI	
Avaliação de disfunção de DAV	
Tumores cardíacos com suspeita de malignidade em programação de cirurgia ou biópsia	
**Situação de atendimento semi-eletivo**	
Detecção de trombo em átrio esquerdo na FA persistente	
Disfunção valvar com apresentação subaguda ou crônica	
Pesquisa de doença arterial coronariana em angina estável	
Doença cardíaca estrutural estável	
Tumores cardíacos provavelmente benignos sem programação de cirurgia ou biópsia	
**Situação de atendimento eletivo**	
Demais condições clínicas	

DAV: dispositivo de assistência ventricular; FA: fibrilação atrial; MINOCA: infarto do miocárdio sem lesões coronarianas obstrutivas; TAVI: implantação percutânea de valva aórtica.

### h. Medicina Nuclear

**Table:** 

**Classificação: Ambiente de contato moderado**
**Risco: Médio Risco**

Como na seção de ecocardiografia, é importante definir o escalonamento de prioridades dos exames de medicina nuclear, observando-se o perfil clínico do paciente. Exames eletivos e semi-eletivos (perfis C e D) devem ser considerados para reagendamento posterior enquanto a transmissão comunitária da COVID-19 esteja ocorrendo de forma sustentada. Esse aspecto é particularmente importante para pacientes de risco para COVID-19, incluindo idade > 60 anos, hipertensão arterial sistêmica, diabetes, doença pulmonar crônica e demais doenças crônicas, um perfil bastante comum entre aqueles que realizam exames de medicina nuclear. Para pacientes com perfil clínico A ou B, em que o exame seja considerado necessário e não substituível, ou em casos selecionados de pacientes com perfil clínico C em que o exame seja necessário, os protocolos de cuidado de nível 2 devem ser recomendados. Considerar sempre os riscos descritos para o teste ergométrico, caso o estresse físico seja aventado. Devido ao baixo grau de evidência, esta recomendação é feita por segurança e outras diretrizes podem recomendar somente o uso de máscara cirúrgica em pacientes sem suspeita de COVID-19. Esta estratégia é particularmente aceitável em casos de COVID-19 curado ou em casos sem sintomas e exame negativo recente. Assim, as orientações abaixo buscam alinhar o planejamento com as diretrizes mundiais:

- **Com o intuito de abreviar o tempo do exame**
- Selecionar o protocolo com a menor duração de tempo de aquisição;
- Considerar o início do protocolo pela fase de estresse e realização do exame em apenas um dia, principalmente em pacientes com baixa probabilidade de isquemia miocárdica;
- Considerar protocolos restritos à imagem de estresse;
- Considerar agente farmacológico com menor tempo de infusão.

- **Com o intuito de reduzir o risco de exposição do profissional de saúde**
- Avaliar rigorosamente o critério para o estresse físico com teste ergométrico para, nesse momento, minimizar seu uso, assim priorizando protocolos com estresse farmacológico;
- Considerar uso de manguitos automáticos de pressão arterial quando disponíveis;
- Considerar manter a vigilância por vídeo durante o teste;
- Em protocolos de estresse com adenosina e dipiridamol, extensores podem ser usados para manter o distanciamento entre a equipe e o paciente;
- O PET com 18F-FDG deve ser considerado para endocardite como uma alternativa ao ETE, que apresenta uma exposição muito alta ao risco de gotículas para os operadores.

Além das medidas implementadas no contexto da pandemia, a rápida evolução e o seu impacto no mundo podem resultar em uma possível escassez ou dificuldade de distribuição de medicamentos e radiofármacos. Sendo assim, um controle de fluxo mais restrito é essencial, principalmente durante o período de alto número de novos casos de COVID-19 ou em situações de possível disrupção da logística de distribuição das medicações.

## 6. Hemodinâmica e Cardiologia Intervencionista

**Table:** 

**Classificação: Contato invasivo de vias aéreas**
**Risco: Muito Alto Risco**

### a. Procedimentos Eletivos

#### Recomendações Gerais

- Pacientes eletivos devem seguir fluxos distintos no setor de hemodinâmica em relação aos casos emergenciais.
- Em serviços com mais de uma sala de intervenção, deve-se manter sala exclusiva para casos eletivos.
- Manter *Heart Team* atuante durante a pandemia e envolvido na fase de reabertura.
- Aplicar o Questionário de Sintomas e Exposição antes da realização de procedimentos intervencionistas eletivos (Suplemento A).
- No início do processo de reabertura, selecionar os pacientes com maior potencial de benefício com a intervenção coronária percutânea ou intervenção em cardiopatia estrutural (Tabela 9).

**Tabela 9:** Pacientes com maior potencial de benefício com a intervenção

Doença coronariana sintomática de difícil manejo clínico
Doença coronariana e achados de alto risco em prova funcional
Doença coronariana e achados anatômicos de alto risco, como lesão obstrutiva grave do tronco da artéria coronária esquerda ou do terço proximal da artéria descendente anterior
Estenose aórtica grave sintomática ou assintomática com FEVE reduzida
Insuficiência mitral em CF III/IV e progressão recente, com queda recente da FEVE, ou nos serviços com programas estabelecidos de tratamento percutâneo da insuficiência mitral por *clip* mitral

CF: classe funcional; FEVE: fração de ejeção de ventrículo esquerdo.

**Figura 3:**
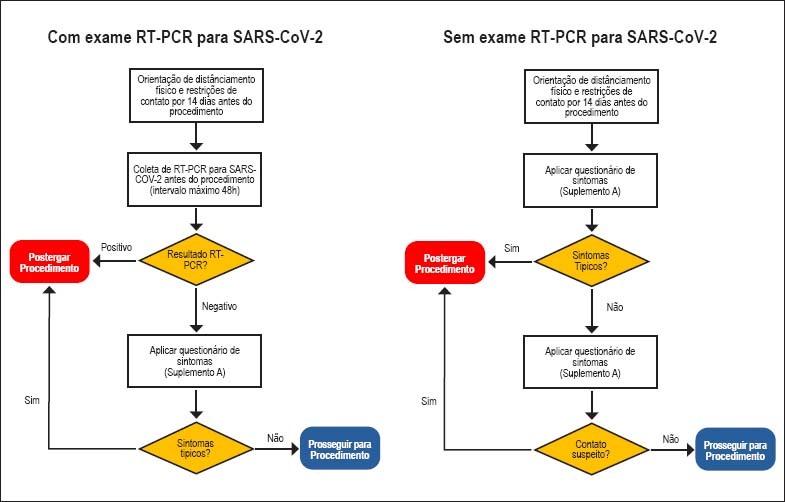
Fluxograma de conduta para procedimentos eletivos de cardiologia intervencionista e eletrofisiologia de acordo com a presença e ausência do exame de RT-PCR para SARS-CoV-2.

Apesar de controversa, a utilização de exames de RT-PCR para diagnóstico de COVID-19 em pacientes assintomáticos que internem no hospital para procedimentos eletivos pode ser considerada adequada para redução de risco de transmissão nosocomial. Nesses casos, pode-se coletar o *swab* nasofaríngeo/orofaríngeo, em caráter ambulatorial, para RT-PCR para SARS-CoV-2 nas 48 horas que antecedem os procedimentos, devendo a coleta ser realizada preferencialmente em regime domiciliar. Essa prática facilita a alocação hospitalar dos pacientes durante a internação, racionaliza a utilização de EPI específico e minimiza os riscos de exposição da equipe. Se a coleta de RT-PCR para SARS-CoV-2 não estiver disponível, sugerimos fluxograma alternativo (Figura 3).

#### Recomendações Gerais

- Obtenção de consentimento informado após esclarecimento dos riscos e benefícios do procedimento no contexto da pandemia.
- Recomenda-se adiar, por no mínimo 14 dias, os procedimentos eletivos em pacientes sintomáticos ou com RT-PCR para SARS-CoV-2 positivo. Em pacientes de perfil clínico C e D, deve-se considerar o risco-benefício de se adiar a realização do procedimento até controle da pandemia.
- Em implantes de valva aórtica transcateter, preconizar a abordagem minimalista com sedação consciente quando factível, priorizando encurtamento do tempo de internação hospitalar e reduzindo a utilização do ETE.

#### Recomendações Pré-procedimento

##### 1. Local com Coleta de RT-PCR Pré-internação de Rotina

- Recomenda-se coletar RT-PCR para SARS-CoV-2 por *swab* nasal e orofaríngeo em prazo máximo de 48 horas antes do procedimento. Preferencialmente oferecer coleta domiciliar.
- Todos os pacientes devem ser orientados quanto à manutenção de distanciamento social e restrição de contatos nos 14 dias que antecedem o procedimento.
- Realizar, mediante contato telefônico ou por meio eletrônico, rastreio de sintomas e exposição (Suplemento A) nas 48 horas que antecedem a realização do procedimento.
- Se houver acompanhante no período de internação, aplicar Questionário de Sintomas e Exposição e solicitar RT-PCR.
- Se o teste de RT-PCR detectar a presença do vírus, aguardar no mínimo 14 dias com melhora dos sintomas há pelo menos 3 dias para reagendar o procedimento. Não é necessário coletar novo RT-PCR para liberação do procedimento, pois o exame poderá permanecer positivo mesmo com vírus inviável.

##### 2. Local sem Realização de RT-PCR Antes da Internação

- Todos os pacientes devem ser orientados quanto à necessidade de isolamento domiciliar e restrição absoluta de contato nos 14 dias que antecedem o procedimento.
- Realizar rastreio de sintomas e de exposição de risco (Suplemento A) antes do exame para obter a melhor segurança.

#### Recomendações Durante o Procedimento

Utilização de EPI de nível 3 (precaução contra contato + aerossóis) para todos os operadores.

#### Recomendações Pós-procedimento

- Encaminhar pacientes para o setor de recuperação anestésica conforme resultado da triagem (área para COVID-19 positiva e área para COVID-19 negativa). Se não for possível, a recuperação anestésica poderá ser realizada na própria sala de procedimento.
- Priorizar alta no mesmo dia ou, caso isso não seja possível, minimizar o tempo de internação.
- Quando a intervenção ou exame for seguida de internação hospitalar, utilizar fluxo hospitalar apropriado e leitos reservados para pacientes sem COVID-19.
- Após a alta, como indicador de segurança, realizar o seguimento dos pacientes quanto ao surgimento de sintomas no período de até 10 dias após o procedimento. Caso o paciente desenvolva sintomas, deve-se realizar a busca de contatos hospitalares do paciente-índice.

### b. Procedimentos de Urgência e Emergência

Com o número elevado de suscetíveis na população, a retomada dos programas de intervenções percutâneas eletivas ocorrerá concomitantemente com as admissões hospitalares relacionadas à infecção por SARS-CoV-2, que reconhecidamente aumenta o risco de fenômenos tromboembólicos e determina múltiplas manifestações cardiológicas, como síndromes coronarianas agudas, miocardites, infarto do miocárdio tipo II e arritmias ventriculares, além de cardiomiopatia de estresse (Síndrome de Takotsubo) desencadeada pela própria doença. Dessa forma, é fundamental a manutenção de um fluxo próprio para atendimento de casos suspeitos ou confirmados de COVID-19, bem como para atendimentos emergenciais de pacientes com síndromes coronarianas agudas com *status* infeccioso desconhecido e sem possibilidade de testagem.

#### Recomendações Gerais

- Reservar uma sala para casos de COVID-19 confirmada ou suspeita e para pacientes de emergência, em serviços com duas salas ou mais.

#### Recomendações Pré-procedimento

- Não retardar o atendimento dos casos de emergência, como infarto agudo do miocárdio (IAM) com supradesnivelamento do segmento ST;
- Pacientes com diagnóstico pré-hospitalar via telemedicina de IAM com supradesnivelamento do segmento ST e pacientes referenciados por outras unidades para angioplastia primária ou angioplastia de resgate devem ser encaminhados diretamente ao setor de hemodinâmica, evitando-se fluxo pelo pronto-atendimento.

#### Recomendações Durante o Procedimento

- Disponibilizar EPI de nível 3 (precaução contra contato + aerossóis) para toda a equipe.
- Manter equipe treinada em técnicas de paramentação e desparamentação.
- Manter equipe médica e equipe multidisciplinar em número reduzido no interior da sala.
- Reduzir materiais sobre as bancadas e checar *tool box* antes dos procedimentos para evitar a abertura da porta.
- Remover armários, quando possível, ou manter suas portas fechadas durante todo o procedimento.
- Em salas sem pressão negativa, minimizar a abertura da porta e utilizar áudio do sistema de comunicação para contato com o meio externo.
- Destinar um técnico no exterior da sala com paramentação de proteção completa para entrega de materiais com aberturas breves da porta;
- Usar sedação de modo parcimonioso para evitar instrumentalização da via aérea.
- Realizar entubação orotraqueal (IOT) com técnica de sequência rápida, quando necessário.
- Em pacientes com comprometimento progressivo da mecânica respiratória, priorizar IOT e evitar utilização de ventilação não invasiva e de cânula nasal de alto fluxo.

#### Recomendações Pós-procedimento

- Ao término do procedimento, encaminhar o paciente para internação em zona específica para COVID-19 confirmada ou para pacientes aguardando RT-PCR para COVID-19.
- Aguardar o transporte dentro da sala de hemodinâmica para evitar contaminação do ambiente de recuperação pós-anestésica.

#### Higienização Pós-procedimento

- Realizar a desparamentação dentro da sala.
- Realizar limpeza terminal da sala de forma rotineira.

## 7. Eletrofisiologia

**Table:** 

**Classificação: Contato invasivo de vias aéreas**
**Risco: Muito Alto Risco**

### a. Procedimentos Eletivos

Devem-se observar as recomendações gerais de reabertura do programa de eletivos descrita anteriormente para os procedimentos invasivos em hemodinâmica, com obtenção de consentimento informado considerando os riscos e os benefícios do procedimento e o contexto da pandemia, observando-se ainda fluxos distintos para os exames eletivos e os atendimentos de urgência/emergência.

Os exames/intervenções com maior grau de urgência (Tabela 9) e maior impacto prognóstico devem ser priorizados para reduzir o risco de morte e prevenir descompensações clínicas.

- Recomenda-se adiar procedimentos eletivos em pacientes sintomáticos ou com RT-PCR positivo recente mesmo que assintomáticos.
- Apesar de controversa, a utilização de exames de RT-PCR para diagnóstico de COVID-19 em pacientes assintomáticos admitidos para procedimentos eletivos em eletrofisiologia pode ser considerada adequada para redução de risco de transmissão nosocomial (Figura 3).

### b. Procedimentos de Urgência e Emergência

Diante de cenários clínicos com risco iminente de descompensação hemodinâmica ou óbito (Tabela 10), pacientes com COVID-19 ou *status* infecioso desconhecido podem ser admitidos para intervenções de emergência. Nesses casos, devem ser observadas as recomendações de segurança da equipe em concomitância.

**Tabela 10:** Classificação dos procedimentos de eletrofisiologia durante a pandemia de COVID-1919

**Situação de atendimento eletivo**
Estudo eletrofisiológico ou ablação de FA, *flutter* ou ablação nodal em pacientes estáveis ambulatoriamente
Estudo eletrofisiológico para avaliar taquiarritmias estáveis
Cardioversão de arritmias estáveis com sintomas tolerados
Fechamento do apêndice atrial esquerdo em pacientes com condição de anticoagulação oral
Tilt-table test
**Situação de atendimento semi-eletivo**
Ablação de taquicardia ventricular refratária e recorrente em terapia medicamentosa
Taquicardias supraventriculares determinando múltiplas visitas ao pronto-atendimento
Ablação de FA, *flutter* ou ablação nodal em pacientes com sintomas recorrentes
**Situação de atendimento de urgência e emergência**
Ablação de taquicardia ventricular por tempestade elétrica refratária à terapia medicamentosa
Síndrome de Wolf-Parkinson-White ou pré-excitação com síncope ou parada cardiovascular
Ablação de FA, *flutter* ou ablação nodal em casos de comprometimento hemodinâmico significativo e refratariedade à terapia medicamentosa ou à cardioversão

FA: fibrilação atrial.

As recomendações de cuidados durante e após o procedimento são semelhantes àquelas apresentadas para serviços de hemodinâmica conforme descrito acima.

## 8. Considerações Especiais Acerca dos Pacientes Envolvidos no Transplante Cardíaco

A COVID-19 tem repercussões específicas para os pacientes envolvidos no contexto do transplante cardíaco, incluindo doadores e receptores, tanto na lista de espera como após o transplante. Como já conhecido, trata-se de uma população de risco aumentado para infecção por SARS-CoV-2 e progressão para doença grave em razão das comorbidades, do contato constante com unidades e profissionais de saúde e da imunossupressão. Por outro lado, trata-se de um perfil de alta adesão às recomendações médicas.^20^ Assim, são necessárias estratégias de prevenção e tratamento direcionadas.^21^

Com relação ao doador, devemos estar atentos à escolha de indivíduos não infectados, reconhecendo que muitos podem ser portadores assintomáticos/pré-sintomáticos/oligossintomáticos e que os testes atuais têm limitações importantes. Assim:

- Recomenda-se realizar o teste RT-PCR para SARS-CoV-2 logo que consentida a doação de órgãos.
- Sempre que disponível, orienta-se a realização de TC de tórax para afastar achados radiográficos suspeitos de infecção.
- Se o resultado do RT-PCR for positivo, seus órgãos não devem ser utilizados para transplante.

Quando internados, os pacientes tanto da fila de espera como aqueles em pós-operatório de transplante:

- Devem ser mantidos em unidades “não COVID-19”, reservando-lhes atendimento por uma equipe multiprofissional que não tenha contato com casos positivos para infecção por SARS-CoV-2.
- Devem ter as visitas presenciais controladas e não recomendadas. A rotina de comunicação com familiares deve ser organizada.

No contexto ambulatorial, deve-se manter a recomendação deste posicionamento:

- Reduzir visitas presenciais para pacientes estáveis e sem sintomas.
- Estimular o telemonitoramento dos níveis séricos dos imunossupressores.
- Postergar rotina de biópsia endomiocárdica em pacientes estáveis.

No paciente receptor de transplante cardíaco, em razão do *status* de imunossupressão sustentada, pode ocorrer manifestação típica (respiratória) e atípica (gastrointestinal) na infecção por SARS-CoV-2. Nesses casos, recomenda-se:

- Considerar reduzir a dose de inibidor de calcineurina (ciclosporina ou tacrolimus).
- Suspender temporariamente os antiproliferativos (micofenolato ou azatioprina).

## 9. Orientações ao Paciente com Fatores de Risco para Infecção por SARS-CoV-2 e suas Repercussões Clínicas

O cardiologista clínico cumpre um papel essencial no cuidado dos pacientes com infecção pelo SARS-CoV-2, devido à alta prevalência de fatores associados a maior risco de repercussões clínicas mais graves no contexto da infecção viral. Assim, o adequado controle dos fatores de risco torna-se objetivo indispensável na continuidade do tratamento, na tomada de novas decisões e na orientação para redução do risco de contágio e de infecção.

### Pacientes com Risco Aumentado para Formas Graves de Infecção por SARS-CoV-2

Na Tabela 6, foram apresentados os fatores de risco para formas graves de COVID-19. Esses pacientes devem ser avisados quanto à sua situação e à necessidade de continuidade do tratamento:

- Não alterar nem suspender o uso das medicações sem antes consultar seu médico.
- Manter um suprimento de medicamentos pelo menos mensal.
- Manter as vacinas atualizadas de acordo com o calendário de imunizações (em especial contra influenza e doença pneumocócica).
- Reforçar a importância de permanecer fisicamente ativo e praticar hábitos saudáveis, inclusive como medida de redução de risco de complicações da COVID-19.^22^ A prática de exercícios físicos dentro das normas de segurança recomendadas para que se evite o contágio por COVID-19 deve ser incentivada.
- O combate ao tabagismo deve ser fortemente recomendado, inclusive em populações de menor risco, como indivíduos jovens, tendo em vista o aumento da chance de complicações.^23^
- Nunca postergar a procura por pronto-atendimento em caso de qualquer sinal de alerta, como dor torácica, dispneia, sintomas de alteração de fala, marcha e/ou força muscular localizada ou outra condição que requeira atendimento imediato.

Tais recomendações visam consolidar a adesão ao tratamento e o controle de fatores de risco cardiovascular (em especial obesidade e tabagismo), uma estratégia que pode agregar valor adicional à redução do risco de complicações da COVID-19.^24^^,^^25^

Além disso, deve-se orientar a manutenção de medidas para diminuição do risco de contágio, tais como:

- Limitar as interações de proximidade física com outras pessoas, sempre que possível.
- Tomar precauções devidas ao interagir com outras pessoas:
  ◦ Distanciamento de pelo menos 2,0 m.
  ◦ Uso de máscara facial de tecido.
- Higiene das mãos com água e sabão ou álcool gel.
- Entrar em contato com seu médico em caso de febre, diarreia ou sintomas respiratórios.
- Em caso de suspeita de COVID-19, contraindicar a automedicação e orientar sobre os riscos.
- Evitar atividades nas quais não é possível tomar medidas de proteção, como situações onde o distanciamento social não pode ser mantido (reuniões em ambiente fechado, eventos).
- Evitar aproximação com outras pessoas que não estejam tomando medidas de proteção.

### Cuidados após a Alta do Paciente Cardiológico e Retorno ao Trabalho após Infecção por SARS-CoV-2

Estudos indicam que até 20% dos pacientes apresentam alguma complicação cardiovascular durante a internação por COVID-19, entre elas arritmias, síndrome coronariana aguda e injúria miocárdica.^21^ Esses pacientes têm apresentação clínica da COVID-19 mais grave e mortalidade três vezes maior. Da mesma forma, esses pacientes podem enfrentar diversos obstáculos no ambiente domiciliar após a alta. Para aqueles com limitações funcionais após a alta, deve-se reforçar a necessidade de reabilitação.

- Física: muitos pacientes receberão alta necessitando de cuidados por limitações respiratórias ou cuidados em área de feridas/pressão. Também serão necessárias intervenções para recuperar a massa muscular e a capacidade funcional naqueles com neuromiopatia do paciente crítico.
- Psicológica e neuropsicológica: como resultado de suas experiências de doença e tratamento, pacientes em recuperação podem desenvolver adversidades psicológicas persistentes ou até mesmo comprometimento cognitivo.
- Socioeconômico: as necessidades e circunstâncias sociais e econômicas dos pacientes foram comumente afetadas pela pandemia. O impacto potencial de mudanças durante o isolamento também deve ser considerado.

Por esse motivo, nos pacientes que apresentaram manifestação cardiovascular grave da doença, deve-se considerar reavaliação na primeira semana após a alta hospitalar para verificação dos sintomas cardiovasculares, adesão medicamentosa e esclarecimento de dúvidas e dificuldades de readaptação às atividades rotineiras, levando-se em conta os procedimentos de reabilitação descritos abaixo. Ainda, o tempo mínimo para o retorno às atividades laborais deve seguir as diretrizes já existentes e levar em conta a funcionalidade após a alta e o tempo de isolamento mínimo necessário.

### Reabilitação Cardiovascular

Estabelecida cientificamente como importante intervenção na prevenção secundária, a reabilitação cardiovascular é uma das medidas com indicação IA pela SBC em diversos contextos de cuidados na doença arterial coronariana, no pós-operatório de cirurgias cardíacas e na insuficiência cardíaca, tendo particular importância no período após a alta de internações por quadros agudos, como é o caso da COVID-19.

No atual momento, onde há necessidade de se cumprirem as diretrizes de distanciamento social e redução de mobilidade, destaca-se a urgência na implementação de modelos eficazes de combinação de atendimento presencial e de telemonitoramento remoto. No âmbito mundial, diferentes meios de comunicação têm sido utilizados nesse processo de reabilitação virtual (por exemplo, telefone/celular, aplicativos para *smartphone*, e-mail, mensagem de texto, páginas da Internet, videoconferências). No entanto, é essencial a avaliação individual do risco-benefício para os atendimentos à distância, como menor intensidade no treinamento físico intensivo, menor apoio social, padrões de treino remoto ainda em elaboração e preocupações de segurança em pacientes com maior risco. A adaptação do ambiente de reabilitação cardiopulmonar durante a pandemia de COVID-19 está detalhada na Tabela 11.

**Tabela 11:** Recomendações para a adaptação dos centros de reabilitação cardiopulmonar para a pandemia da COVID-19

Aplicar o questionário de rastreamento de sintomas e contato (Suplemento A) e cancelar o atendimento presencial em caso de qualquer resposta positiva
Uso obrigatório de máscaras cirúrgicas pelo paciente e pela equipe durante toda a permanência no centro de reabilitação
Manter distância mínima de 2,0 m, sempre que possível, durante o uso de aparelhos (cicloergômetro, esteira)
Organizar sessões individuais ou reduzir o número de pacientes por atendimento o máximo possível
Desinfecção sistemática do material utilizado antes e depois de cada atividade
Utilizar programas mais curtos, concentrando os esforços nos componentes principais de cada paciente atendido
Substituir, sempre que possível, as sessões presenciais por avaliação e monitoramento remotos, orientando os pacientes de acordo com o equipamento e com o meio de comunicação mais adequado para o momento (telefone, mensagens de texto, e-mails, consultas por vídeo-chamada, plataformas e aplicativos dedicados)
Promover estratégias especiais para a maioria dos imunocomprometidos, como pacientes transplantados cardíacos
Interromper as atividades comunitárias que não respeitem as regras de distanciamento social

## 10. Segurança dos Pacientes e Profissionais de Saúde na Pandemia

Proteger pacientes e profissionais de saúde em todos os níveis deve ser o objetivo principal na retomada das atividades durante a pandemia. Estudos já demonstraram a alta taxa de contaminação entre profissionais da saúde, além do potencial papel desse grupo na disseminação da COVID-19, como superdisseminadores, tanto no ambiente de trabalho quanto na comunidade.

A higienização das mãos e o uso adequado do EPI são imprescindíveis para minimizar os riscos de contaminação dos trabalhadores de saúde pelo SARS-CoV-2. Desse modo, é de suma importância que toda a equipe receba treinamento sobre a utilização correta do EPI, com atenção especial à paramentação e à desparamentação, que devem ser padronizadas para reduzir o risco de contaminação. Toda a equipe deve receber capacitação e demonstrar habilidade para colocação, uso, retirada e descarte correto e seguro do EPI.^3^^,^^26^

A orientação de utilização do EPI específico deve ser baseada no risco biológico a que os profissionais estarão expostos durante as atividades e deve atender às seguintes recomendações:

- Regularização junto aos órgãos certificadores e à Anvisa;
- Utilização adequada, higienização ou descarte periódico, conforme recomendações técnicas;
- Inspeção, reparo e substituição de acordo com instruções do fabricante.

No entanto, além da segurança física da equipe, deve-se considerar também a segurança legal, psicológica, econômica e informacional.

### a. Segurança Física

Assegurar a integridade física do profissional de saúde envolvido é uma das principais metas na implantação de medidas de retorno às atividades. Além do treinamento contínuo de toda a equipe, da aplicação diária do Questionário de Sintomas e Exposição em toda a equipe e do rastreamento de contatos de casos confirmados, são essenciais o fornecimento e o uso racional e sistemático de EPI.

#### Seleção da Equipe no Retorno ao Trabalho

Como os serviços voltarão de forma gradual à sua capacidade total, é importante selecionar para o retorno inicialmente os profissionais de saúde com menor risco e considerar a adequação do risco aos ambientes de trabalho onde o profissional é alocado.

Primeiramente, evidências sugerem que os profissionais mais jovens e sem fatores de risco sejam priorizados em ambientes de “contato próximo” e “invasivo de vias aéreas”, onde o risco ocupacional de infecção por SARS-CoV-2 é maior. Por outro lado, profissionais idosos ou de meia-idade com fatores de risco devem ser encorajados, até momento oportuno, a manter-se em ambiente de atendimento remoto e/ou com distanciamento físico rigoroso. Para os demais casos, pode-se seguir a Tabela 12.

**Tabela 12:** Classificação das prioridades a serem consideradas para retorno da equipe de trabalho

	Jovem, sem fator de risco	Meia-idade ou jovem com fator de risco*	Idoso ou meia-idade com fator de risco*
Contato invasivo de vias aéreas	1	2	3
Contato próximo	1	2	3
Contato moderado	1	2	2
Sem contato / Remoto	1	1	1

Prioridade 1 – retornar imediatamente Prioridade 2 – retornar após esgotados os profissionais de saúde em prioridade 1 Prioridade 3 – Não retornar a princípio, salvo extrema necessidade

* Os fatores de risco estão listados na Tabela 4

#### Medidas de Controle de Transmissão e Isolamento de Casos Suspeitos

O profissional de saúde que apresentar sintomas sugestivos de infecção por SARS-CoV-2 deve ser prontamente afastado. A seguir, deve-se realizar a investigação dos contatos dos 4 dias anteriores ao início dos sintomas. Os contatos devem ser monitorados, orientados a fazer quarentena por 10 dias do último contato e, caso disponível, realizar *swab* nasofaríngeo para pesquisa do vírus por RT-PCR.

#### Critérios para Retorno às Atividades Laborais após COVID-19

##### • Casos suspeitos

O profissional de saúde com quadro clínico suspeito de COVID-19 (RT-PCR negativo ou não coletado) poderá retornar às atividades após preenchidos ambos os critérios:

1. Pelo menos 3 dias (72 horas) após critério de recuperação clínica, definida como:

- Resolução da febre sem o uso de antitérmicos.
- Melhora dos sintomas respiratórios (tosse, falta de ar) E

2. Pelo menos 10 dias desde o início dos sintomas.

##### • Casos confirmados

◦ *Sintomáticos*

O profissional de saúde com confirmação de COVID-19 (RT-PCR positivo) poderá retornar às atividades após preenchidos ambos os critérios:

1. Pelo menos 3 dias (72 horas) após critério de recuperação clínica, definida como:

- Resolução da febre sem o uso antitérmicos.
- Melhora dos sintomas respiratórios (tosse, falta de ar), E

2. Pelo menos 10 dias desde o início dos sintomas.

◦ *Assintomáticos*

Devido à ausência de sintomas, não é possível avaliar onde esses indivíduos estão no curso de sua doença. Assim, caso haja confirmação por RT-PCR positivo em assintomático, o critério para liberação do isolamento será:

➢ Pelo menos 10 dias do resultado do teste positivo.

Para pacientes assintomáticos confirmados por testes sorológicos, não há orientação clara sobre o retorno às atividades após testes positivos. No entanto, levando-se em conta a segurança do ambiente de trabalho, deve-se considerar a estratégia mais segura. Logo, sugere-se:

➢ Sorologia IgM ou IgA reagente ou IgG/IgM reagentes – retorno após 10 dias do teste.
➢ Sorologia IgG reagente – sem necessidade de afastamento.

Aconselha-se consultar especialistas em doenças infecciosas para definir tempo de retorno ao trabalho para os indivíduos que possam permanecer transmissores por mais de 10 dias (por exemplo, imunocomprometidos).

#### Residência Médica e Complementação especializada

Os estagiários são uma parte importante dos programas e serviços de cardiologia em boa parcela do país. Durante a pandemia do COVID-19, muitos deles foram transferidos de seus estágios de imagem para o atendimento clínico de pacientes com COVID-19 em hospital e unidades de terapia intensiva.

No retorno às atividades, sugere-se que os iniciantes sejam deslocados para divisões onde sua inexperiência não aumente o tempo de exposição da equipe ao paciente. A educação precisa ser revisada buscando novos métodos de aprendizado, incluindo abordagens de aprendizado baseadas em videoconferências e treinamento remoto.

### b. Segurança Legal

Pacientes admitidos devem assinar previamente termo de consentimento, de preferência contendo informações de que estão cientes que o procedimento está sendo realizado durante epidemia de COVID-19, com riscos inerentes ao procedimento e ao momento excepcional. Também são recomendados documentação e armazenamento dos questionários diários de sintomas e exposição tanto dos profissionais de saúde como dos pacientes, para caso haja necessidade futura, assim como documentação por escrito de rotinas de precaução contra transmissão consideradas pela equipe responsável pelo atendimento.

#### Profissionais de Saúde em Situação de Risco

Como citado anteriormente no tópico de *Seleção da equipe no retorno às atividades*, alguns profissionais não retornarão de imediato ao trabalho. Nesses casos de afastamento (Tabelas 6 e 12) ou durante o período de sintomas, isolamento e reabilitação em caso de infecção por SARS-CoV-2, faz-se necessária uma rede de apoio entre os especialistas para encaminhamento imediato dos pacientes sob sua custódia.

### c. Segurança Psicológica

À medida que a situação de pandemia continua, são necessárias estratégias para apoiar psicologicamente os profissionais de saúde, em especial o grupo mais suscetível a sofrimento psicológico. O suporte psicológico pode incluir serviços de aconselhamento e desenvolvimento de sistemas de apoio entre colegas. São também parte do plano:^27^^,^^28^

- Monitorar constantemente a equipe quanto ao bem-estar, em especial se for necessário trabalhar em jornadas prolongadas ou quando remanejados para áreas desconhecidas.
- Facilitar o acesso aos serviços de saúde mental e de apoio psicossocial.
- Manter busca ativa de profissionais em condições psicológicas comprometidas e em situação de *burnout.*
- Exigir o *feedback* periódico dos colaboradores.
- Fornecer atualizações de informações precisas a todos os funcionários.
- Considerar rodízios das funções de maior estresse físico/emocional com as de menor estresse.

### d. Segurança Econômica

No momento atual, os custos crescentes com o cuidado ao paciente com COVID-19, aliados à receita reduzida, pressionam financeiramente as instituições de saúde tanto no ambiente público como na iniciativa privada. É essencial que o comitê diretor mantenha atualizado de maneira constante o planejamento financeiro, negociando os repasses públicos no ambiente do Sistema Único de Saúde, ainda mais sobrecarregado nessa pandemia, e assegurando-se de acordo com a previsão de receitas quando na saúde suplementar. Ênfase particular deve ser dada ao aumento de custos associados a adaptação ambiental, uso de EPI e redução da capacidade de atendimento para garantir o distanciamento físico dos pacientes durante o fluxo no serviço de saúde.

### e. Segurança Informacional

O combate a notícias falsas e o oferecimento de uma literatura de saúde apropriada aos pacientes são missões do cardiologista e da equipe multidisciplinar. A SBC tem emitido informes técnicos orientados ao cardiologista e aos pacientes. A Organização Mundial de Saúde e entidades governamentais têm mostrado sua preocupação sobre a “infodemia” (com a propagação de notícias falsas) e seus impactos sobre a saúde física e psicológica dos pacientes.

É de responsabilidade da equipe de saúde manter-se atualizada e fornecer aos pacientes informações claras, objetivas e embasadas em fontes seguras para evitar a propagação de informações incorretas, incompletas, mal interpretadas ou falsas.

## 11. Suplemento A

Questionário de Sintomas e Exposição para ser aplicado antes da realização de procedimentos intervencionistas eletivos

**Questionário de sintomas:**

**Nos últimos 14 dias você apresentou algum desses sintomas?**

Sintomas maiores (basta 1 para suspeita):

□ Febre
□ Tosse
□ Falta de ar
□ Confusão mental
□ Perda da sensação de gosto / cheiro

Sintomas menores (são necessários 2 para suspeita):

□ Fadiga/cansaço
□ Diarreia
□ Nariz escorrendo
□ Náusea e/ou vômitos
□ Dor de garganta
□ Dor de cabeça
□ Conjuntivite
□ Outro:_________________________

**Questionário de Exposição:**

1. Nos últimos 14 dias você teve contato, por mais de 15 minutos e a uma distância menor que 2,0 m, com alguma pessoa diagnosticada ou identificada pelo médico como suspeita de COVID-19?

□ SIM □ NÃO

2. Nos últimos 14 dias você esteve internado em algum serviço de saúde?

□ SIM □ NÃO

3. Se profissional de saúde: Você esteve em contato sem uso de EPI com pacientes com suspeita ou confirmação de COVID-19?

□ SIM □ NÃO
