# Supplementary material for: Posicionamento – Protocolo de Reconexão dos Serviços de Cardiologia com os Pacientes Durante a Pandemia de COVID-19 – 2020
Source: Arq Bras Cardiol. 2020 Oct 13;115(4):776–99. [Article in Portuguese] doi: 10.36660/abc.20201004 (PMC8386987; doi:10.36660/abc.20201004)
Supplement: Translation

# Translation: Statement – Protocol for the Reconnection of Cardiology Services with Patients During the COVID-19 Pandemic – 2020

**Authors:** Marcio Sommer Bittencourt, Giuliano Generoso, Pedro Henrique M. Craveiro de Melo, Driele Peixoto, Érique José Farias Peixoto de Miranda, Evandro Tinoco Mesquita, Andréa Araujo Brandão, José Francisco Kerr Saraiva, Silvio Henrique Barberato, Fernando Bacal, Marcelo Antônio Cartaxo Queiroga Lopes

**Affiliations:** 1 São PauloSP Brazil Hospital Universitário da Universidade de São Paulo (USP), São Paulo, SP – Brazil; 2 São PauloSP Brazil Hospital Sírio Libanês, São Paulo, SP – Brazil; 3 São PauloSP Brazil Instituto do Câncer do Estado de São Paulo (ICESP), São Paulo, SP – Brazil; 4 São PauloSP Brazil Bayer Pharmaceuticals SA, São Paulo, SP – Brazil; 5 NiteróiRJ Brazil Universidade Federal Fluminense (UFF), Niterói, RJ – Brazil; 6 Rio de JaneiroRJ Brazil Centro de Ensino e Treinamento Edson de Godoy Bueno / UHG, Rio de Janeiro, RJ – Brazil; 7 Rio de JaneiroRJ Brazil Faculdade de Ciências Médicas da Universidade do Estado do Rio de Janeiro (FCM/UERJ), Rio de Janeiro, RJ – Brazil; 8 campinasSP Brazil Sociedade Campineira de Educação e Instrução, campinas, SP – Brazil; 9 CuritibaPR Brazil CardioEco Centro de Diagnóstico Cardiovascular, Curitiba, PR – Brazil; 10 São PauloSP Brazil Instituto do Coração do Hospital das Clínicas da Faculdade de Medicina da Universidade de São Paulo, São Paulo, SP – Brazil; 11 São PauloSP Brazil Hospital Israelita Albert Einstein, São Paulo, SP – Brazil; 12 João PessoaPB Brazil Hospital Alberto Urquiza Wanderley, João Pessoa, PB – Brazil

**Development:** Brazilian Society of Cardiology

**Norms and Guidelines Council (2020-2021):** Brivaldo Markman Filho, Antonio Carlos Sobral Sousa, Aurora Felice Castro Issa, Bruno Ramos Nascimento, Harry Correa Filho, Marcelo Luiz Campos Vieira

**Norms and Guidelines Coordinator (2020-2021):** Brivaldo Markman Filho

**Table:** 

The report below lists declarations of interest as reported to the SBC by the experts during the period of the development of these update, 2020.	
Expert	Type of relationship with industry
Andréa Araujo Brandão	FINANCIAL DECLARATION A - ECONOMICALLY RELEVANT PAYMENTS OF ANY KIND MADE TO (i) YOU, (ii) YOUR SPOUSE/PARTNER OR ANY OTHER PERSON LIVING WITH YOU, (iii) ANY LEGAL PERSON IN WHICH ANY OF THESE IS EITHER A DIRECT OR INDIRECT CONTROLLING OWNER, BUSINESS PARTNER, SHAREHOLDER OR PARTICIPANT; ANY PAYMENTS RECEIVED FOR LECTURES, LESSONS, TRAINING INSTRUCTION, COMPENSATION, FEES PAID FOR PARTICIPATION IN ADVISORY BOARDS, INVESTIGATIVE BOARDS OR OTHER COMMITTEES, ETC. FROM THE BRAZILIAN OR INTERNATIONAL PHARMACEUTICAL, ORTHOSIS, PROSTHESIS, EQUIPMENT AND IMPLANTS INDUSTRY: - Servier: Lectures - Libbs: Hypertension - Novartis: Heart failure - EMS: Hypertension - Sandoz: Hypertension - Abbott: Hypertension OTHER RELATIONSHIPS FUNDING OF CONTINUING MEDICAL EDUCATION ACTIVITIES, INCLUDING TRAVEL, ACCOMMODATION AND REGISTRATION IN CONFERENCES AND COURSES, FROM THE BRAZILIAN OR INTERNATIONAL PHARMACEUTICAL, ORTHOSIS, PROSTHESIS, EQUIPMENT AND IMPLANTS INDUSTRY: - Servier: Hypertension
Driele Peixoto	Nothing to be declared
Érique José Farias Peixoto de Miranda,	FINANCIAL DECLARATION A - ECONOMICALLY RELEVANT PAYMENTS OF ANY KIND MADE TO (i) YOU, (ii) YOUR SPOUSE/PARTNER OR ANY OTHER PERSON LIVING WITH YOU, (iii) ANY LEGAL PERSON IN WHICH ANY OF THESE IS EITHER A DIRECT OR INDIRECT CONTROLLING OWNER, BUSINESS PARTNER, SHAREHOLDER OR PARTICIPANT; ANY PAYMENTS RECEIVED FOR LECTURES, LESSONS, TRAINING INSTRUCTION, COMPENSATION, FEES PAID FOR PARTICIPATION IN ADVISORY BOARDS, INVESTIGATIVE BOARDS OR OTHER COMMITTEES, ETC. FROM THE BRAZILIAN OR INTERNATIONAL PHARMACEUTICAL, ORTHOSIS, PROSTHESIS, EQUIPMENT AND IMPLANTS INDUSTRY: - Bayer: Xarelto OTHER RELATIONSHIPS EMPLOYMENT RELATIONSHIP WITH THE BRAZILIAN OR INTERNATIONAL PHARMACEUTICAL, ORTHOSIS, PROSTHESIS, EQUIPMENT AND IMPLANTS INDUSTRY, AS WELL AS ANY EMPLOYMENT RELATIONSHIP WITH HEALTH INSURANCE COMPANIES OR MEDICAL AUDIT COMPANIES (INCLUDING PART-TIME JOBS) IN THE YEAR TO WHICH YOUR DECLARATION REFERS: - Bayer
Evandro Tinoco Mesquita	OTHER RELATIONSHIPS EMPLOYMENT RELATIONSHIP WITH THE BRAZILIAN OR INTERNATIONAL PHARMACEUTICAL, ORTHOSIS, PROSTHESIS, EQUIPMENT AND IMPLANTS INDUSTRY, AS WELL AS ANY EMPLOYMENT RELATIONSHIP WITH HEALTH INSURANCE COMPANIES OR MEDICAL AUDIT COMPANIES (INCLUDING PART-TIME JOBS) IN THE YEAR TO WHICH YOUR DECLARATION REFERS: - UnitedHealth Group
Fernando Bacal	FINANCIAL DECLARATION A - ECONOMICALLY RELEVANT PAYMENTS OF ANY KIND MADE TO (i) YOU, (ii) YOUR SPOUSE/PARTNER OR ANY OTHER PERSON LIVING WITH YOU, (iii) ANY LEGAL PERSON IN WHICH ANY OF THESE IS EITHER A DIRECT OR INDIRECT CONTROLLING OWNER, BUSINESS PARTNER, SHAREHOLDER OR PARTICIPANT; ANY PAYMENTS RECEIVED FOR LECTURES, LESSONS, TRAINING INSTRUCTION, COMPENSATION, FEES PAID FOR PARTICIPATION IN ADVISORY BOARDS, INVESTIGATIVE BOARDS OR OTHER COMMITTEES, ETC. FROM THE BRAZILIAN OR INTERNATIONAL PHARMACEUTICAL, ORTHOSIS, PROSTHESIS, EQUIPMENT AND IMPLANTS INDUSTRY: - Novartis: Entresto
Giuliano Generoso	Nothing to be declared
José Francisco Kerr Saraiva	Nothing to be declared
Marcelo Antônio Cartaxo Queiroga Lopes	Nothing to be declared
Marcio Sommer Bittencourt	FINANCIAL DECLARATION A - ECONOMICALLY RELEVANT PAYMENTS OF ANY KIND MADE TO (i) YOU, (ii) YOUR SPOUSE/PARTNER OR ANY OTHER PERSON LIVING WITH YOU, (iii) ANY LEGAL PERSON IN WHICH ANY OF THESE IS EITHER A DIRECT OR INDIRECT CONTROLLING OWNER, BUSINESS PARTNER, SHAREHOLDER OR PARTICIPANT; ANY PAYMENTS RECEIVED FOR LECTURES, LESSONS, TRAINING INSTRUCTION, COMPENSATION, FEES PAID FOR PARTICIPATION IN ADVISORY BOARDS, INVESTIGATIVE BOARDS OR OTHER COMMITTEES, ETC. FROM THE BRAZILIAN OR INTERNATIONAL PHARMACEUTICAL, ORTHOSIS, PROSTHESIS, EQUIPMENT AND IMPLANTS INDUSTRY: - Sanofi: Research - EMS: Lectures Novo Nordisk: Lectures C - PERSONAL RESEARCH FUNDING PAID BY THE BRAZILIAN OR INTERNATIONAL PHARMACEUTICAL, ORTHOSIS, PROSTHESIS, EQUIPMENT AND IMPLANTS INDUSTRY: - Sanofi: Research
Pedro Henrique M. Craveiro de Melo	Nothing to be declared
Silvio Henrique Barberato	Nothing to be declared

## 1. Introduction and General Concepts

The pandemic resulting from the novel coronavirus (SARS-CoV-2) infection, named COVID-19, emerged in Wuhan, China, in December 2019.^1^^,^^2^ Since then, millions of people around the world have been affected and hundreds of thousands have died.

The virus spreads mainly from person to person,^1^^,^^2^ directly via the airways or indirectly through contact with contaminated surfaces and objects. Respiratory infections occur through the transmission of virus-filled droplets (> 5 *μ*m, approximate extension of 1.5 m) or aerosols (≤ 5 *μ*m, approximate extension of 8 m) exhaled by infected individuals.^3^^,^^4^ Recent data have suggested the possibility of airborne transmission. The contact of the contaminated respiratory droplets, eliminated when infected persons breath, talk, sneeze and cough, with the ocular, oral and nasal mucosa of susceptible individuals results in SARS-CoV-2 transmission. With the dissemination of COVID-19 and the fast increase in the number of cases, studies have shown the viability of the virus in the environment as well as the role played by contaminated surfaces in SARS-CoV-2 hospital spread.^5^ Based on droplet transmission, the Centers for Disease Control and Prevention of the United States have recommended the practice of physical distancing of at least 1.5 m and hand hygiene to reduce viral spread.

The COVID-19 clinical presentation can range from mild/moderate illness, which affects 80% of the cases, to more severe illness, which affects the other 20% with findings that in Brazil are comparable to those of the severe acute respiratory syndrome (SARS).^6^ The most sensitive symptoms of mild to moderate illness are dry cough and fever, which might or not be present at disease onset, while the most specific symptoms are olfactory and gustative disorders.^7^^,^^8^ The SARS is defined as a influenza-like illness (ILI) associated with dyspnea/respiratory distress OR persistent chest pressure OR O**2** saturation <95% on room air OR bluish coloration of lips and face, with tachypnea, cyanosis and severe pulmonary impairment.

Because of the virus spread potential, the severe clinical presentation of some cases and the lack of a specific viral treatment, or even a vaccine, nonpharmacological measures have been the most effective interventions to contain disease spread.^9^^,^^10^ Such non-pharmaceutical interventions are aimed at reducing the person-to-person viral transmission by limiting the contact between infected and susceptible individuals, either by increasing the distance between people or reducing the intensity and duration of the contact or by using measures and physical or chemical devices that help prevent the person-to-person viral spread.

The non-pharmaceutical interventions can be as follows:

- Physical distancing: consists in keeping a minimum physical distance of 2.0 m between individuals, some examples being school dismissal, closure of crowded places, and travel restrictions.
- Measures of transmission blockade: hand hygiene with water and soap or alcohol, respiratory etiquette, and use of face masks or other physical contact barriers.
- Identification and isolation of cases of suspected or confirmed COVID-19: maintain individual isolation.
- Quarantine of contacts: active tracing of contacts of confirmed cases and their household confinement, in addition to clinical course monitoring of suspected cases.

This pandemic is a daunting challenge to the population. The current humanitarian crisis is due not only to COVID-19 itself, but also to the collateral damage associated with the following: the delay and reduction in the care provided to other diseases in emergency settings; the reduction in the access to care provided to chronic illnesses in outpatient settings; and the higher exposure to risk factors for the development of other diseases (sedentary lifestyle, obesity, anxiety, and emotional stress). Moreover, economic losses pile up, as do the psychological impact and burnout associated with the COVID-19 pandemic. The decrease in the proper delivery of general healthcare can occur mainly in two circumstances. First, there is a reduction in the number of routine medical consultations and tests. This disruption in the chronic patients’ care might lead to acute decompensation of some conditions, such as arterial hypertension, diabetes, and heart failure. Similarly, other severe consequences to face might be related to the reduction in the search for urgency and emergency healthcare for time-sensitive circumstances, such as acute coronary syndromes, acute heart failure, and stroke, for which delay compromises outcomes.^11^^-^^13^

To minimize those adversities, the Brazilian Society of Cardiology (SBC) has compiled recent evidence, which is limited and sometimes grounded in expert opinions or preliminary reports, to create structured guidance aimed at reconnecting physicians to patients for a planned reopening of cardiological services. Such guidance is intended for risk reduction for not only patients and patient companions, but also healthcare professionals involved in the clinical activities of Cardiology.

The recommendations presented in this document are based on evidence available at the time of its elaboration and on expert opinions. The knowledge about COVID-19 progresses fast and dynamically, thus the protocols for the safe return of medical care, as well as of invasive and non-invasive procedures, are constantly being updated. This project was conceived by the SBC to be a source of reference material for its associates. The recommendations presented, however, should not be used as the sole base to define local protocols, and other updated sources should be considered as the knowledge in the field evolves.

This position paper was aimed at aligning the following demands:

- To minimize the risk of SARS-CoV-2 transmission among patients, healthcare personnel and others involved in the patient’s care.
- To early identify patients with suspected COVID-19 and conduct triage to assign proper levels of care, to reduce the risk of complications from COVID-19 and ongoing cardiovascular diseases, and to reduce the risk of viral transmission.
- To provide patients and healthcare professionals with safe and reliable information on COVID-19-related care.
- To reduce the negative impact from the lack of outpatient management of pre-existing conditions on the emergency and hospital admission settings.
- To optimize the use of personal protective equipment (PPE).

Thus, to deliver excellent care, ensuring the safety of healthcare professionals and patients with cardiovascular disease, we should consider the following assumptions:

- Because the clinical presentation varies, the definition of the presence or absence of SARS-CoV-2 infection might not be possible based only on initial clinical assessment. Clear screening protocols should be used to minimize the traffic of patients with suspected COVID-19 in the healthcare setting, except for urgency or emergency care. When necessary, that traffic should occur with the lowest possible risk of contamination.
- All healthcare professionals should undergo continuous training regarding good practices, institutional protocols, and patient care flowcharts.
- The delivery of emergency care should follow the same protocol of the care provided to patients with confirmed/suspected COVID-19, since COVID-19 cannot be ruled out timely for the proper care in the emergency setting, which should continue to operate at full capacity.
- Elective and semi-elective procedures will be resumed after thorough planning, but should be restricted to limited capacity, with continuous reassessment by the steering committee, always respecting authorizations and occasional traffic restrictions, and reopening of medical services defined by competent authorities.
- Separate pathways for traffic, with access routes to diagnostic equipment clearly indicated using adequate signage to the transportation services, patients and other workers who do not belong to the medical sector, aiming at minimizing contact and exposure. For individuals with suspected or confirmed COVID-19 requiring a test or procedure, the healthcare flows and pathways, as well as the corresponding waiting areas, should be set up apart from the flows and pathways for those without suspected SARS-CoV-2 infection.
- Reduction in the traffic and exposure of healthcare workers (receptionists, and hygiene and administrative professionals).

Finally, the hierarchy of the healthcare flows in different areas of Cardiology requires the definition of the patient’s clinical profile, of the physical distancing between the patient and the healthcare team, of the type of their contact during the delivery of care, in addition to the level of urgency for the treatment of heart diseases.

The reopening time of Cardiology services should align with institutional policies and follow the recommendations of competent authorities. Important considerations include the local incidence of patients with SARS-CoV-2 infection, the trend in the number of disease cases, and the institutional resources available, including facilities, human resources, and PPE supply chains. For example, if the PPE storage is limited, the transitory suspension of elective care delivery might be necessary to prevent PPE shortage in the urgency and emergency care.

As the Brazilian regions tend towards the COVID-19 epidemic control after its transforming impact on healthcare services, that guidance might relax restrictions on a regional basis. Therefore, monitoring patients and healthcare workers is crucial to identify the progression of possible local transmission and the need to increase the response level inside medical institutions, particularly those with hospital beds. Thus, the medical community must remain on the alert and attentive to the evolving character of the recommendations both in the state or municipal level and even in their offices, clinics, and hospitals.

## 2. Healthcare and Patient’s Clinical Profiles, and Healthcare Environment

### a. General Guidance to all Healthcare Settings

To resume healthcare activities safely, it is essential that all medical services define a local plan elaborated with the active participation of the medical staff, of the professionals with experience in infection control and patient’s safety, as well as of all healthcare professionals involved. Thus, the following initial measures, applicable to all healthcare levels, are proposed:

- Activities should resume with limited capacity, for example, 25% of the pre-COVID-19 maximum capacity, enabling the controlled implementation of interventions and assessment of healthcare flow. After obtaining an appropriate flow, the healthcare volume can be increased in a staggered way. However, reaching the pre-COVID-19 flow volumes should not be expected during the period of relaxation of restrictions because of the adaptations required. The potential impact of these measures on the financial sustainability of the health service should be considered.
- The care of more symptomatic patients and those with illnesses most likely to decompensate within the next few weeks or months should be prioritized, lowering the chance of hospitalizations and complications in the short and medium term, and helping fight the overload of the hospital admission system.
- Aiming at ensuring physical distancing, adaptations in the physical structure, such as flooring demarcation, are required in association with the installation of physical barriers in the workplace, such as acrylic or glass panels.
- Ensure that hygiene and cleaning materials, such as alcohol gel, tissue papers and hand soap, are readily available and easily reached, in addition to ensuring the proper disposal of residues.
- Elaborate visual reminders, such as banners and posters, which should be made available to patients digitally or displayed at the entrance of the service and at strategic places disclosing key information on hand hygiene, respiratory etiquette, and COVID-19 major signs and symptoms.
- Make sure all healthcare personnel wear proper PPE continuously during the entire time of care delivery (Figure 1).
- Make sure all patients and patient companions older than 2 years wear a mask, and, for those who do not, provide one at the time of triage. It is worth emphasizing that some healthcare settings with proper characteristics, such as hospitals, might have specific regulation enforcing the use of disposable surgical masks. These specifications should be considered by the health services when defining which mask the patient is required to wear.
- For bigger-size medical services with larger teams of workers, a reopening committee should be constituted for the continuous discussion of the adjustments required in the interventions and healthcare volume to reduce the risk of transmission.
- On the day before the consultation or test, the Screening Questionnaire of Symptoms and Exposures (Appendix A) should be applied via email or telephone contact. All patients should complete that questionnaire verbally (telephone) or in writing (e-mail or printed).
- Upon arrival for healthcare, all patients should have their body temperature measured.
- When a consultation or test is required for a patient with suspected or confirmed COVID-19, a separate room should be reserved for it.
- In the hospital setting, barriers should be placed for outpatients, creating ‘clean’ entrances and exits, that is, preventing contact with inpatients.
- For urgency and emergency care (definition in section 2b), where neither previous screening nor healthcare delay is possible, apply objectively, after standard cardiac assessment, the Screening Questionnaire of Symptoms and Exposures to define the epidemiological status.

**Figure 1:** Guidance on PPE according to the risk levels. For level 2, goggles can be replaced with face shield. At level 3, the face shield is mandatory and cannot be replaced by goggles. Adapted from GVIMS/GGTES/ANVISA.

In case of any positive answer to that questionnaire (Appendix A):

Make sure the patient wears a surgical mask.

- Keep the patient in a separate waiting room, with doors closed and, if possible, adequate signage.
- Keep physical distancing of 2.0 m.
- The supervisor and medical care provider should be informed, and all healthcare personnel involved should be wearing proper PPE.
- Urgency and emergency services should define a specific healthcare flow according to the local structure available.

### b. Definition of the Cardiological Care Profile According to its Urgency

Defining the healthcare protocol requires considering the patient’s clinical condition and its urgency. Therefore, four care profiles were defined according to the patients’ clinical characteristics and risk of worsening due to postponing the care:

- Profile A: emergency care setting: when the care, assessment and intervention should be performed within the next minutes or hours.
- Profile B: urgent care setting: when the care, assessment and intervention should be performed within the next days.
- Profile C: semi-elective care setting: when the care, assessment and intervention should be performed within the next weeks, ideally within 3 months.
- Profile D: elective care setting: when the care, assessment and intervention are not deemed necessary in the short term and could be postponed for more than 3 months.

Table 1 shows the major clinical presentations of the cardiovascular diseases according to the above classification of healthcare required.

**Table 1:** Major clinical presentations of cardiovascular diseases according to the urgency of the healthcare required

**Emergency healthcare setting**
ST-elevation myocardial infarction
High-risk acute coronary syndrome
Refractory ventricular arrhythmia
AF due to ventricular pre-excitation syndrome
Acute aortic syndromes
Corrections of cardiac anatomical dysfunctions leading to cardiogenic shock
Dysfunction of an intra- or extracorporeal MCAD
Bradyarrhythmias and tachyarrhythmias with hemodynamic repercussion
Cardiac tamponade
Acute pulmonary edema
**Urgency healthcare setting**
Moderate-risk acute coronary syndrome
Low-risk acute coronary syndrome
Significant valve anatomical dysfunction, symptomatic
AF and recurrent supraventricular tachycardia with clinical repercussion
NYHA functional class IV decompensated heart failure
Urgent heart transplant
High-risk cardiac tumors
Severe congenital heart disease, symptomatic
Severe hypertriglyceridemia > 1000 mg/L
Dysfunction of any component of the CIED
CIED at the end of battery life
**Semi-elective healthcare setting**
Stable angina
Uncontrolled hypertension
NYHA functional class III decompensated heart failure
Uncontrolled diabetes
Moderate valve anatomical dysfunction
Aortic aneurysm
Significant valve anatomical dysfunction, asymptomatic
Patients assessed for MCAD implantation
New cardiac anatomical change in a previously healthy patient
CIED with battery requiring non-urgent replacement
**Elective healthcare setting**
Other cases

AF: atrial fibrillation; CIED: cardiac implantable electronic device; MCAD: mechanical circulatory assistance device; NYHA: New York Heart Association.

### c. Definition of the Patient’s Clinical Profile (COVID-19 Status)

The clinical presentation compatible with suspected or confirmed COVID-19 should consider the definition of FLS and SARS as shown in Table 2.

**Table 2:** Definition of influenza-like illness (ILI) and severe acute respiratory syndrome (SARS)

Classification	Clinical characteristics
ILI	Respiratory symptoms, such as cough, runny nose, sore throat, with or without fever*
SARS	O**2** saturation <95% on room air AND/OR respiratory rate ≥24 bpm

* Fever may not be present in some cases, such as elderly and immunocompromised patients, when the clinical assessment should be taken into consideration.

The clinical criterion, however, does not allow to establish the etiology, and other agents should enter in the differential diagnosis, based on epidemiological evidence, laboratory tests, and radiological findings.

Regarding the SARS-CoV-2 infection, a patient’s clinical profile can be as follows:

- *With suspected COVID-19*: case suspected of FLS or SARS based on presumptive clinical, radiological or laboratory criterion.
- *With confirmed active COVID-19*: case suspected of FLS or SARS with SARS-CoV-2 infection confirmed by use of a definitive laboratory method (positive RT-PCR for SARS-CoV-2) or IgM serology AND:
  ◦ within 10 days from symptom onset or from the test date, if asymptomatic; OR
  ◦ within 3 days from the last COVID-19-related symptom.
- *With confirmed resolved COVID-19*: confirmed COVID-19 with evidence of improvement of clinical findings, defined as absence of fever for >3 days and improvement of the respiratory symptoms (cough, shortness of breath) AND:
  ◦ at least 10 days have passed since the first symptoms OR
  ◦ positive IgG serology with clinical history compatible with previous COVID-19
- *Contact of a suspected or confirmed case*: an individual who came into contact (workplace, household) within the last 14 days with another classified into one of the three clinical profiles above.
- *Asymptomatic individual without recent contact with a case*: an individual with neither symptom suggestive of SARS-CoV-2 infection within the last 10 days nor contact with a suspected or confirmed case within the last 14 days.
- *COVID-19 ruled out*: Case suspected of FLS or SARS without laboratory confirmation of SARS-CoV-2 infection during the diagnostic window of opportunity OR laboratory confirmation of another etiological agent, such as influenza virus or respiratory syncytial virus. In highly suspected cases, it might be necessary to repeat the RT-PCR test in 48 hours due to its limited sensitivity.

### d. Definition of the Healthcare Environment

The above described clinical profiling is useful not only to define the urgency the medical care requires, but also its structural model of approach. Whenever possible, the risk of exposure of healthcare personnel, patients and patient companions should be minimized, prioritizing a safer, even though effective, care. Thus, the healthcare environment was classified according to the distance and contact duration between patients and healthcare workers:

- *Environment I: Remote care*, where there is no physical contact between the patient and the healthcare team.
- *Environment II: Moderate-contact setting*, where there is in-person contact between the patient and the healthcare team, with short-time exposure (< 15 minutes) and/or greater physical distancing (> 1.5 m).
- *Environment III: Close-contact setting*, there is close or prolonged physical contact between the patient and the healthcare team (>15 minutes, in a closed environment).
- *Environment IV: Invasive contact and potential airway manipulation*, there is either direct contact with the patient or patient’s airway manipulation, and consequent exposure to aerosols.

To make it clearer, Table 3 depicts examples of several environments of routine cardiological care. It is worth emphasizing that diagnostic/procedural reports, whenever possible, should be elaborated and issued remotely.

**Table 3:** Classification of Cardiology care environments according to person-to-person contact

**Cardiology care environments**
**Remote care**
Decision-making support systems
Teleconsulting, including telephonic guidance
Tele Regulation
Telediagnosis and remotely issued reports
Teleducation
**Moderate-contact environment**
Parking lot (valet parking)
Receptionists and administrative workers
Security professionals
**Close-contact environment**
In-person medical appointment
Presence of healthcare personnel in nuclear medicine tests
Presence of healthcare personnel in imaging tests (CT, MRI, echocardiography)
Presence of healthcare personnel in cardiology graphical methods
**Invasive contact and potential airway manipulation**
Oroscopy and physical exam
Transesophageal echocardiography
Invasive procedures in electrophysiology
Invasive procedures in interventional cardiology

CT, computed tomography; MRI, magnetic resonance imaging.

## 3. COVID-19 Precautionary Measures and Confinement

Four levels of occupational risk from exposure to SARS-CoV-2 have been distinguished: extremely high, high, intermediate, and low (Figure 2). The occupational risk assessment depends on the distance and duration of contact between the professional and the patient, on the type of procedure performed and its risk of aerosol generation, in addition to the patient’s clinical profile regarding the SARS-CoV-2 infection.

**Figure 2:** Pyramid of occupational risk for COVID-19 (adapted from Occupational Safety and Health Administration - OSHA).^14^

Therefore, in addition to standard precautionary measures, the following should be implemented by the healthcare services:

◦ Precautionary measures against contact
◦ Precautionary measures against droplets
◦ Precautionary measures against aerosols

Some aerosol-generating procedures are as follows: tracheal intubation or aspiration, non-invasive mechanical ventilation, cardiopulmonary resuscitation, manual ventilation before intubation, nasotracheal sample collection, bronchoscopy. For the healthcare in which these aerosol-generating procedures are routine or potentially necessary, precautionary measures against droplets should be replaced with precautionary measures against aerosols.

Thus, the necessary PPE for healthcare delivery to suspected or confirmed cases of COVID-19 are as follows:

### a. Standard Precautionary Measures

Instituted for all patients and comprise:

- Hand hygiene.
- Masking – during the COVID-19 pandemic, face masks should be worn for standard precaution, as detailed below for level 1 PPE.

If there is risk of exposure to blood or secretions:

- Wear procedural gloves.
- Wear goggles and gown.

Dispose PPE waste into appropriate containers.

### b. Precautionary Measures Against Contact + Droplets:

- Surgical face mask.
- Googles or face shield.
- Gown with minimum weight of 30 g/m^2^.
- Procedural gloves.

### c. Precautionary Measures Against Contact + Aerosols:

- N95/PPF2 respirator or equivalent.
- Face shield.
- Fluid-resistant gown.
- Hair cap.
- Procedural gloves.

In addition to aerosol-generating procedures, when managing patients with sputum, bleeding, vomiting or diarrhea, the healthcare professional should wear a fluid-resistant gown. The rational use of PPE in healthcare services is required, and because of the risk of PPE shortage, the recommendations on PPE use may change due to contingency plans during the COVID-19 pandemic.

### d. Recommendation on PPE Use According to the Procedure Complexity and the Patient’s Clinical Profile (Figures 1 and 2, Table 4)

**Table 4:** Definition of the PPE usage level according to risk

Risk	Exposure type	Healthcare type	PPE level
Extremely high risk	Exposure to patients with confirmed or suspected COVID-19	Oroscopy and ophthalmoscopy TEE, exercise stress testing Invasive procedures in electrophysiology and interventional cardiology	Level 3
Invasive contact of airways	Aerosol-generating procedures		
High risk	Exposure to patients with confirmed or suspected COVID-19	Nuclear medicine tests Imaging tests (CT, MRI, TTE) Graphical methods in cardiology	Level 2
Close-contact environment			
Intermediate risk	Frequent and in-person contact	Receptionists Administrative workers Parking lot In-person appointment	Level 1
Moderate-contact environment			
Low risk	No contact with patients	Tele Regulation Telediagnosis Teleducation	No specific PPE
Remote care

CT: computed tomography; MRI: magnetic resonance imaging; TEE: transesophageal echocardiography; TTE: transthoracic echocardiography. For in-person consultations of patients with suspected or confirmed COVID-19, level 2 PPE should be worn. Some guidelines consider that imaging tests and graphic methods, except for exercise test, require the use of only surgical mask, particularly when the likelihood of active COVID-19 is low, when COVID-19 is resolved or in cases with neither symptoms nor recent contact and a recent negative test for COVID-19.

**Level 1 PPE**: For intermediate risk, moderate-contact environment.

#### Standard Precautionary Measures

Indicated for NON-invasive procedures when performed electively AND in patients without suspected COVID-19.

- Professionals involved in activities favoring contact between one another greater than 2.0 m can wear a cloth face mask.
- If the 2.0 m physical distancing from patient is not guaranteed, a surgical mask should be worn during the activities (Table 5).
- Physical barriers, such as acrylic plaques and flooring demarcation, should be installed to favor distancing of at least 2.0 m (Table 5).

**Table 5:** Standard precaution according to distancing

Precaution type	Standard
Professionals > 2.0 m from patient	Cloth face mask
Professionals < 2.0 m from patient	Surgical mask

A cloth face mask should not be used by healthcare personnel during care delivery.

**Level 2 PPE**: For high risk, close-contact environment.

#### Precautionary Measures Against Contact + Droplets

Indicated for NON-invasive procedures when performed in an emergency situation OR in patients with confirmed OR suspected COVID-19.

**Level 3 PPE**: For extremely high risk, invasive contact of airways.

#### Precautionary Measures Against Contact + Aerosols

Indicated for invasive and **aerosol-generating** procedures performed:

- electively AND in patients with **no** suspected COVID-19.
- in an emergency situation OR in patients with confirmed OR suspected COVID-19.

Note: For invasive procedures, replace the PPE (gloves, gowns) with properly sterilized equipment.

It is worth emphasizing that cloth face masks are not PPE, thus they should not be worn by healthcare or ancillary personnel in situations requiring the use of either a surgical mask (during healthcare or direct contact, in which the minimum 2.0 m distance between one another cannot be kept) or an N95/PFF2 respirator (during aerosol-generating procedures).

### e. Surface cleaning and disinfection

There is no differentiated recommendation for surface cleaning and disinfection after contact with cases of suspected or confirmed COVID-19. However, it is mandatory that healthcare services revise their operational procedures for cleaning and disinfection of environment and surfaces to ensure better practices and increased periodicity of cleaning.^14^^,^^15^^,^^16^

- All specific equipment in cardiological management should be cleaned at the end of the workday and professionals should be wearing PPE to prevent contact with infected materials.
- Monitors and surfaces of the ultrasound device can be covered with a disposable plastic wrap to reduce the risk of contamination and facilitate cleaning.
- After a patient with suspected or confirmed COVID-19 leaves the procedure room, the equipment and all areas that the patient and healthcare professionals had contact with should be cleaned/disinfected with usual sanitizers.

## 4. Strategic Approach in the Healthcare Environment

### a. In-person Healthcare Environment

**Table:** 

**Classification: Environment II (moderate contact)**
**Risk: Low to intermediate**

In-person healthcare is provided in the out-of-hospital environment, which comprises basic healthcare units, outpatient speciality clinics, hospital outpatient clinics, clinics, and medical offices. These are an essential part of the health system response, because of the potential increase in cardiovascular outcomes in the medium-term due to the deficit in the medical care for that population. Adaptations of the healthcare protocol should include the following changes in routine care:^16^

#### Scheduling

- Appointments should be scheduled remotely (telephone or online), using dedicated digital platforms or message apps.
- Fewer appointments should be scheduled and at longer intervals, allowing a patient to leave the healthcare facility, including the waiting room, before the next one arrives, minimizing contact between one another.
- The triage of patients with respiratory symptoms should be performed during scheduling and upon appointment confirmation by using the Screening Questionnaire of Symptoms and Exposures (Appendix A).
- The use of a face mask by the patient and patient companion should be advised and required regardless of either the presence of symptoms or their responses to the Screening Questionnaire of Symptoms and Exposures.
- The patient should be advised to attend the appointment alone, whenever possible. When strictly necessary, indicate that only one companion is allowed, and this person should also complete the same Screening Questionnaire of Symptoms and Exposures.

#### Physical Structure/ Waiting Room

- Keep the environment ventilated (air conditioning with ventilation, which ensures air exchange, or opened windows).
- Downsize the reception personnel that has physical contact with patients.
- Install physical barriers in the reception to reduce contact, such as acrylic plaques and flooring demarcation to remind people to maintain at least 2.0 m between one another.
- Display visual reminders at the service entrance and strategic places disclosing key information on hand hygiene, respiratory etiquette, and signs and symptoms of suspected COVID-19.
- Provide tissue paper for nasal hygiene or respiratory etiquette in the waiting room, in addition to foot-pedal trash cans for discarding tissues and paper towels.
- Provide dispensers with alcoholic preparations for hand hygiene and sinks with liquid soap dispensers, paper towel and its support, touchless garbage can.
- Encourage waiting in an open or external environment, observing the minimum 2.0 m physical distancing.
- Demarcate the minimum 2.0 m distance between the seats.
- Provide disposable cups for water and coffee.
- Remove shared objects from the room, such as magazines and newspapers.
- Advise on hand hygiene before and after completing the forms, using pens, and paying with credit card. Clean the credit card payment machine and, to facilitate the process, the device can be covered with a disposable plastic wrap and cleaned after each use.
- Provide surgical masks for patients with respiratory symptoms and instruct them on the correct mask wearing during their entire stay in the unit, if the care is strictly necessary. Patients requiring elective or semi-elective care, however, should return home for individual confinement and investigation for COVID-19, and a new appointment rescheduled for later. Masks should be replaced whenever moist or dirty.
- A patient with respiratory symptoms should be referred to a separated room or area, isolated from other patients.
- Increase the cleaning and disinfection frequency of the environment, objects and higher-contact surfaces.
- Neither carpets soaked in sanitizing solutions nor disinfection booths at the entrance of the service are recommended due to lack of documented efficacy.
- No product should be sprayed onto patients, patient companions or healthcare workers because of the high risk of intoxication and lack of scientific evidence for its use.

#### Patient Companion

- Advise patients to come to the appointment alone.
- If a companion is required for a patient who needs supervision or for any other reason, allow the presence of only one person who does not belong to the risk group (Table 6).
- On the day prior to the appointment, apply the Screening Questionnaire of Symptoms and Exposures to the patient companion.
- Patients requiring elective or semi-elective care with companions who have symptoms or had recent contact with suspected or confirmed COVID-19 cases should not attend a medical appointment.
- Patient companions should follow all the instructions and recommendations provided to patients.

**Table 6:** Risk group assessment for screening the patient companion

Age > 65 years
Obesity
Systemic arterial hypertension
Diabetes mellitus
Smoking
Heart failure
Chronic obstructive pulmonary disease
Chronic kidney disease
Sickle cell anemia
Continuous immunosuppression (transplants, HIV infection, oncological diseases, chronic use of immunosuppressants)
Asthma (moderate to severe)
Cerebrovascular disease
Pregnancy
Liver disease

### b. Healthcare Via Telemedicine

**Table:** 

**Classification: Environment I (remote)**
**Risk: None**

Telemedicine plays a fundamental role in the rational return to healthcare activities and was detailed in the 2019 Brazilian Society of Cardiology Guideline on Telemedicine in Cardiology.^17^ In the context of that guideline, we should emphasize the teleconsultation and telemonitoring concepts and highlight that their use is recommended, except for urgency and emergency patients, who require in-person care. In addition to the general recommendation for telemedicine use, it is worth noting that remote care should be preferred when the healthcare provider belongs to the risk group.

- All telemedicine consultations must take place with synchronous image and sound, in addition to equipment that ensures patient privacy and confidentiality, as well as proper recording of information in a patient’s medical record.
- Telemedicine can be used routinely for pre-consultation by the physician, who should assess the patient’s need for an in-person visit, define the risk of the patient’s clinical findings, refer the patient to a hospital, when necessary, or even solve the case providing complete healthcare via telemedicine.
- Remote healthcare should be preferably provided with digital certification and electronic signature, ensuring originality of the information, as well as safety for both physician and patient.

### c. Remote Follow-up

**Table:** 

**Classification: Environment I (remote)**
**Risk: None**

- Telemonitoring of necessary vital signs and test results can be performed via other remote access media. Some examples of telemonitoring: follow-up of heart failure symptoms and blood pressure levels; telemetry of cardiac implantable electronic device; remote control of laboratory test results, such as kidney function, anticoagulation etc.
- Telemonitoring can and should be used for the remote follow-up of patients to reduce the number of in-person medical visits and, thus, the traffic of patients, whenever possible.

## 5. Strategic Approach in the Context of Non-Invasive Tests

### a. Graphical Methods in Cardiology

The complementary graphical methods in Cardiology can be classified according to the environment they are performed in and the distance between patients and healthcare professionals required, as follows:

*Remote Healthcare*

- Home Blood Pressure Monitoring (HBPM).

*Moderate-contact Environment*

- Electrocardiography at rest.
- Holter.
- Ambulatory Blood Pressure Monitoring (ABPM).

*Close-contact Environment*

- Tilt-table test.

Similarly to the instructions provided to other Cardiology areas, appointments should be scheduled remotely and at programmed hours to reduce the number of people at the same time in the waiting room. The Screening Questionnaire of Symptoms and Exposures should be applied on the day before the procedure for both the patient and the patient companion, which should only be present if essential and not belonging to the risk group for COVID-19 (Table 6).

In the specific context of the graphical methods, remote diagnostic media should be encouraged, such as remote access and real-time telediagnosis, which reduce not only the number of healthcare professionals exposed but the duration of their exposure as well. When these tests cannot be delayed, the instructions include:

- Issue electrocardiogram, Holter, ABPM and HBPM reports remotely.
- Prioritize the use of HBPM over ABPM, whenever possible.
- Follow the institutional protocols of distancing and hygiene/cleaning for the facility and the return of Holter and ABPM devices.
- To assess myocardial ischemia, consider an alternative to exercise testing, with association of imaging techniques and pharmacological stress to reduce the personnel exposure to droplets and aerosols.

### b. Exercise Stress Testing

**Table:** 

**Classification: Invasive contact of airways**
**Risk: Extremely high**

Exercise testing is a valuable tool, considering its widespread use, and the major method for assessing myocardial ischemia in several services across the country. However, one should be cautious during the period of sustained community transmission of COVID-19, because of the higher risk of disease spread resulting from the increase in the patient’s respiratory rate and emission of droplets during the test, as well as the long length of stay in the testing room, which is a closed environment. Therefore, elective and semi-elective tests should be considered on a case-by-case basis. Postponing an exercise test is recommended during the phase of higher community transmission of SARS-CoV-2.

In the rare situation in which exercise testing is deemed necessary for a patient with recent suspected or confirmed COVID-19, it should be performed observing the specific level 3 (contact precaution + aerosols). Due to the fact that scientific evidence is still uncertain regarding this type of exposure, there is a possibility of a change in the recommendation in future publications.

With regard to the tilt test, although there is prolonged exposure by the health professional, there is no increase in respiratory work, and it can be inferred that the risk of transmission is not so increasing. Therefore, level 3 precaution is recommended.

- When undergoing an exercise test, patients should wear a surgical mask during all the procedure.^18^
- Other people should not be present in the testing room (relatives, coaches etc.).
- Automatic blood pressure cuffs should be used whenever available.
- The healthcare team should wear proper PPE according to their distance from the patient, specific level 2 precaution.
- During the entire procedure, the healthcare team should stay at least 2.0 m away from the patient.
- The testing room should be actively ventilated and ideally at least 60 minutes between individual tests should be observed for each treadmill, allowing enough time for proper hygiene of the equipment.
- All equipment used for the test should be thoroughly cleaned between each patient assessed.

### c. Echocardiography

The reopening of echocardiography services should contemplate the already described measures regarding remote scheduling, larger interval between appointments, recommendations for patient companions, environment cleaning and hygiene, and physical distancing. Because of the close contact between the equipment operator and patients required during image acquisition, postponing elective and semi-elective tests should be considered during the sustained community transmission period, mainly for patients of the risk groups (Table 7).

**Table 7:** Priority regarding eligibility for echocardiography testing

**Urgency and emergency healthcare environment**
Recent clinically relevant cardiovascular symptoms (FC III or IV heart failure, syncope of cardiac origin, chest pain, arrhythmias)
Recent procedure requiring urgent follow-up
Arrhythmias after device implantation
Pericardial effusion
Post-operative assessment in cardiac surgery
Initial assessment before beginning oncological drug treatment (chemotherapy/immunotherapy)
Suspected infective endocarditis with high pre-test probability
**Semi-elective healthcare environment**
Asymptomatic patient with chronic cardiac disease that requires monitoring
Assessment of stable heart valve disease (aortic and mitral stenosis or regurgitation)
Pulmonary hypertension
Disease progression after intervention (recurrent coarctation, duct stenosis)
Non-cardiological therapy requiring continuous monitoring
Pulmonary artery systolic pressure estimation in patients on specific therapy
Assessment of rejection after heart transplant
Treatment for Kawasaki disease
Follow-up assessment of the VAD function in stable patients
Non-urgent pre-operative echocardiography
**Elective healthcare environment**
Routine follow-up of chronic diseases: hypertension, coronary artery disease, annual assessment of aorta disease or of prosthetic valve function (normal function on the previous test and no new symptom)

FC: functional class; VAD: ventricular assistance device.

### d. Transthoracic Echocardiography (TTE)

**Table:** 

**Classification: Close-contact environment**
**Risk: High**

- In the initial phase of reopening, schedule high-priority tests first and then intermediate-priority ones, according to local epidemiological status and reopening success;
- Apply in advance the Screening Questionnaire of Symptoms and Exposures and repeat triage before the procedures;
- Healthcare personnel should wear proper PPE.

### e. Transesophageal Echocardiography (TEE)

**Table:** 

**Classification: Invasive contact of airways**
**Risk: Extremely high**

The general considerations for performing TEE follow the same principles already described. However, additional precaution is recommended because of the aerosol-generating potential related to the cough reflex in patients with unprotected airways. Therefore, proper complete and universal PPE should be worn, and thorough cleaning of the testing room and equipment performed.

Ideally, aerosol-generating procedures should be carried out in a respiratory isolation unit with negative pressure and HEPA (High Efficiency Particulate Arrestance) filter. When such unit is not available, the patient should be placed in an individual room with doors closed, and the number of healthcare professionals should be restricted during those procedures. Because of the high risk TEE poses, its indication should be assessed on an individual basis during the COVID-19 pandemic.

#### Recommended PPE

All professionals in contact with the patient’s airways and the support team in the procedure room should take specific level 3 precaution (precaution against contact + aerosols).

#### Equipment Cleaning

Detailing the TEE equipment cleaning is beyond the scope of this document. Other cleaning and disinfection protocols provided by hospital infection control services and institutional technical rules should be followed routinely.

### f. Stress Echocardiography

**Table:** 

**Classification: Close-contact environment**
**Risk: High**

Echocardiography performed with physical stress promotes the same respiratory rate changes described for exercise testing, compounded by the fact that the operator cannot keep proper distance from the patient. Thus, physical stress should be considered rather an exception and not applied routinely until the COVID-19 community transmission is properly controlled. An alternative is performing stress echocardiography with pharmacological agents or using other imaging methods when necessary. In exceptional cases, when physical stress echocardiography must be performed, specific level 3 precaution (precaution against contact + aerosols) should be taken by all professionals in the testing room.

Stress echocardiography with dobutamine or other pharmacological agents should be the preferred alternative during the pandemic, and all precautionary measures recommended for performing an echocardiography at rest should be observed.

#### Patients with Confirmed or Suspected COVID-19

In patients with acute findings and diagnosed with confirmed or suspected COVID-19, echocardiography should be performed only in case of urgency and emergency, when the result of the test is expected to really impact on the clinical management. The use of portable devices is recommended, with special attention being paid to team protection.

- Plan echocardiography in advance to analyze only the windows necessary for decision making.
- Use the time at the patient’s side only to acquire images and videos, leaving measurements to be taken later with a dedicated software.
- Reassign training professionals and less experienced echocardiographers to non-COVID-19 areas to minimize test duration.
- The healthcare team should wear the recommended PPE according to their proximity to the patient, specific level 3 precaution (precaution contact + aerosols).

### g. Computed Tomography (CT) and Magnetic Resonance Imaging (MRI) of the Heart

**Table:** 

**Classification: Moderate-contact environment**
**Risk: Intermediate**

Radiology services and their imaging rooms worldwide have not been projected contemplating restrictive measures against the transmission of infectious diseases. However, imaging tests are essential tools for the diagnosis and treatment of COVID-19 and its complications.

The previously mentioned initial measures related to remote appointment scheduling, longer intervals between appointments, recommendations regarding patient companions, healthcare environment cleaning/disinfection, and physical distancing must be observed.

In the adaptation process for service reopening under the current uncommon conditions, patients with suspected or confirmed COVID-19 should undergo tests preferably using devices/equipment dedicated to their clinical profile, to prevent crossed contamination between infected and non-infected individuals. This is particularly important, because CT is frequently used in the investigation of cases with suspected or confirmed COVID-19. When that is not possible, it is necessary to define, in the equipment available, time slots dedicated to the clinical profile ‘suspected or confirmed COVID-19’, preferably at the end of the workday. Because SARS-CoV-2 remains viable on several surface types and environmental conditions, the imaging room must be cleaned and disinfected after being used for each patient with suspected or confirmed COVID-19 and before acquiring images from a patient without suspected or confirmed COVID-19, and that cleaning should follow institutional protocols.

Elective and semi-elective tests should be rescheduled during the most intense COVID-19 community transmission period. Urgency and emergency tests should be considered according to the clinical need and the expectation of management definition based on test results.

For in-patients, cardiovascular imaging can be used to replace invasive tests or tests that involve airway manipulation, such as TEE and coronary angiography. Thus, in some selected cases, cardiac CT can be used to search for left atrial appendage thrombus, or coronary tomography angiography used in non-ST- elevation acute coronary syndrome. For clinical profile A or B patients, for whom the tests are deemed necessary and irreplaceable, or in selected clinical profile C patients who need the tests, level 2 precaution is recommended. Because of the low level of evidence, this recommendation is made for the purpose of safety and other guidelines might recommend the use of only surgical mask for the care of patients with no suspected COVID-19. This strategy is particularly acceptable in cases of resolved COVID-19 or in cases with no symptom and a recent negative test for COVID-19.

Cardiac MRI, because of the long time it requires to be performed, and thus, longer exposure of the healthcare personnel, should be indicated for patients with suspected or confirmed COVID-19 only exceptionally, such as for the differential diagnosis between myocarditis, Takotsubo syndrome, and myocardial infarction with no obstructive coronary artery disease (MINOCA). Table 8 details the indications and priority levels for CT and MRI.

**Table 8:** Indications for cardiac computed tomography / magnetic resonance imaging during COVID-19 pandemic

**Urgency healthcare environment**
Non-ST-elevation acute coronary syndrome – differential diagnosis, to rule out:	
	Coronary artery disease
	Acute myocarditis
	MINOCA
	Takotsubo syndrome
Exclusion of pulmonary embolism (triple rule-out protocols)	
Detection of left atrial thrombus in AF in an in-patient	
Heart valve dysfunction with acute decompensation	
Suspected valvular endocarditis	
Pre-TAVI planning	
Assessment of VAD dysfunction	
Surgery or biopsy programming for suspected malignant cardiac tumors	
**Semi-elective healthcare environment**	
Detection of left atrial thrombus in persistent AF	
Subacute or chronic heart valve dysfunction	
Investigation of coronary artery disease in stable angina	
Stable structural cardiac disease	
Probably benign cardiac tumors with no surgery or biopsy programming	
**Elective healthcare environment**	
Other clinical conditions	

AF: atrial fibrillation; MINOCA: myocardial infarction with no obstructive coronary artery disease; TAVI: transcatheter aortic valve implantation; VAD: ventricular assistance device.

### h. Nuclear Medicine

**Table:** 

**Classification: Moderate-contact environment**
**Risk: Intermediate**

Similarly to echocardiography, it is important to define the priorities of nuclear medicine tests, observing the patient’s clinical profile. Elective and semi-elective tests (profiles C and D) should be considered for later rescheduling during COVID-19 sustained community transmission. This is particularly important for patients with risk factors for COVID-19, such as age > 60 years, systemic arterial hypertension, diabetes, chronic pulmonary disease, and other chronic illnesses, characteristics frequently found among those undergoing nuclear medicine tests. For clinical profile A or B patients, for whom the tests are deemed necessary and irreplaceable, or in selected clinical profile C patients who need the tests, level 2 precaution is recommended. The risks described for exercise testing should always be weighed when physical stress is considered. Because of the low level of evidence, this recommendation is made for the purpose of safety and other guidelines might recommend the use of only surgical mask for the care of patients with no suspected COVID-19. This strategy is particularly acceptable in cases of resolved COVID-19 or in cases with no symptom and a recent negative test for COVID-19. Thus, the following instructions are intended to align test planning with international guidelines:

- **To shorten test duration**
- Select the protocol with the shortest acquisition duration;
- Consider initiating the protocol at the stress phase and performing the test in one single day, mainly for patients with low probability of myocardial ischemia;
- Consider protocols restricted to stress imaging;
- Prefer pharmacological agents requiring the shortest time of infusion.

**• To reduce the healthcare professional’s risk of exposure**
- Strictly assess the physical stress criterion for exercise testing to minimize its use during the pandemic, prioritizing protocols with pharmacological stress;
- Consider using automatic blood pressure cuffs, when available;
- Consider remote video surveillance during the test;
- In stress protocols with adenosine and dipyridamole, extenders can be used to maintain the distance between professionals and patient;
- For endocarditis, 18F-FDG PET should be considered an alternative to TEE, which determines extremely high exposure to the risk of droplets for device operators.

In addition to the measures implemented in the context of the COVID-19 pandemic, its rapid progression and high impact can result in shortage or difficult distribution of medications and radiotracers. Thus, stricter control of their flow is essential, mainly when the number of new COVID-19 cases is high or in situations of possible disruption of the logistics of medication distribution.

## 6. Catheterization Laboratory and Interventional Cardiology

**Table:** 

**Classification: Invasive contact of airways**
**Risk: Extremely high**

### a. Elective Procedures

#### General Recommendations

- In the cardiac catheterization laboratory (cath lab), elective patients should follow different flows from those of emergency patients.
- In services with more than one intervention room, an exclusive room for elective cases should be kept.
- Keep the Heart Team active during the COVID-19 pandemic and involved in the reopening phase.
- Apply the Screening Questionnaire of Symptoms and Exposures before performing elective interventional procedures (Appendix A).
- At the beginning of the reopening process, select the patients with the highest potential for benefiting from percutaneous coronary intervention or from structural heart disease intervention (Table 9).

**Table 9:** Patients with the highest potential for benefiting from cardiac interventions

Symptomatic coronary artery disease of difficult clinical management
Coronary artery disease and high-risk findings on functional testing
Coronary artery disease and high-risk anatomical findings, such as severe obstruction of the left main coronary artery or of the anterior descending artery proximal third
Symptomatic or asymptomatic severe aortic stenosis with reduced LVEF
Mitral regurgitation in FC III/IV and recent progression, with recent drop in LVEF, or in services with established programs for percutaneous treatment of mitral regurgitation with mitral valve clipping

FC: functional class; LVEF: left ventricular ejection fraction.

Although controversial, the use of RT-PCR testing for COVID-19 diagnosis in asymptomatic patients admitted for elective procedures can be considered adequate to reduce the risk of hospital transmission. In such cases, the nasopharyngeal/oropharyngeal swab for RT-PCR testing can be collected within the 48 hours preceding the procedure, preferably at the patient’s home. This practice facilitates the patients’ hospital allocation during admission, rationalizes the specific PPE use, and minimizes the risks of exposing healthcare personnel. If the RT-PCR is not available, we suggest the alternative flowchart (Figure 3).

**Figure 3:** Flowchart for managing elective procedures of interventional cardiology and electrophysiology according to SARS-CoV-2 RT-PCR testing availability.

#### General Recommendations

- Obtain the patient’s informed consent after clarifying the risks and benefits of performing the procedure during the COVID-19 pandemic.
- Elective procedures in symptomatic patients or those with positive RT-PCR for SARS-CoV-2 should be postponed for at least 14 days. In clinical profile C and D patients, the risk-benefit of delaying the procedure until COVID-19 pandemic control should be considered.
- For transcatheter aortic valve implantation, minimalist approach with conscious sedation, when feasible, is recommended, prioritizing shortening the length of hospital stay and reducing the use of TEE.

#### Preprocedural Recommendations

##### 1. When Routine Pre-admission RT-PCR Testing is Available

- The collection of nasal and oropharyngeal swab for SARS-CoV-2 RT-PCR testing within the 48 hours preceding the procedure is recommended, and preferably at the patient’s home.
- All patients should be instructed to keep social distancing and restrict contacts in the 14 days preceding the procedure.
- By use of telephone or electronic contact, trace symptoms and exposure (Appendix A) in the 48 hours preceding the procedure.
- If a patient companion is required, apply the Screening Questionnaire of Symptoms and Exposures and ask for RT-PCR testing.
- It the RT-PCR test detects the presence of the virus, wait at least 14 days with symptom improvement for at least 3 days to reschedule the procedure. It is not necessary a new RT-PCR testing to approve the procedure, because the result might remain positive even with non-viable virus.

##### 2. When Pre-admission RT-PCR Testing is Not Available

- All patients should be instructed to keep home isolation and absolute contact restriction in the 14 days preceding the procedure;
- Trace symptoms and exposure risk (Appendix A) before the procedure to improve safety.

#### Procedural Recommendations

All operators must use level 3 PPE (precaution against contact + aerosols).

#### Postprocedural Recommendations

- Refer patients to the post-anesthesia recovery room according to the triage result (COVID-19 positive or COVID-19 negative areas). If that is not possible, post-anesthesia recovery should happen in the procedure room.
- Prioritize hospital discharge on the same day, and, if not possible, minimize the length of stay.
- When the intervention or exam is followed by hospital admission, use proper hospital flow and beds reserved for patients without COVID-19.
- After discharge, as an indicator of safety, follow patients up, regarding symptom onset, up to 10 days after the procedure. If the patient develops symptoms, search for the in-hospital contacts of the patient-index.

### b. Urgency and Emergency Procedures

With the elevated number of susceptible individuals in the population, the reopening of programs of elective percutaneous interventions will occur concomitantly with admissions related to SARS-CoV-2 infection, which is known to increase the risk for thromboembolic phenomena and to determine multiple cardiac manifestations, such as acute coronary syndromes, myocarditis, type II myocardial infarction and ventricular arrhythmias, in addition to stress cardiomyopathy (Takotsubo syndrome) triggered by the disease itself. Thus, it is essential to maintain a specific flow for the management of suspected or confirmed COVID-19 cases, as well as for the emergency healthcare of patients with acute coronary syndromes and unknown infectious status and no possibility of testing.

#### General Recommendations

- Reserve one room for confirmed or suspected COVID-19 cases and for emergency patients in services with at least two rooms available.

#### Preprocedural Recommendations

- Do not delay healthcare for emergency cases, such as ST-elevation acute myocardial infarction (AMI).
- Patients with the pre-hospital diagnosis of ST-elevation AMI via telemedicine and those referred from other units for primary angioplasty or rescue angioplasty should be sent directly to the cath lab, avoiding visit to the emergency unit.

#### Procedural Recommendations

- Provide level 3 PPE (precaution against contact + aerosols) for all healthcare personnel.
- Maintain the team trained in the techniques to put on and remove the PPE;
- Reduce the number of individuals in the medical and multidisciplinary teams inside the procedure room to the lowest possible.
- Reduce the materials on the stands and check the toolbox before the procedure to avoid opening the door.
- Remove cabinets, whenever possible, or maintain their doors closed during the entire procedure.
- In rooms without negative pressure, avoid opening the door and use the audio communication system for external contact.
- Assign one technologist wearing proper PPE to be outside the procedure room to deliver materials while briefly opening the door.
- Use sedation parsimoniously to prevent airway management.
- Perform rapid sequence orotracheal intubation (OTI), when necessary.
- In patients with progressive impairment of respiratory mechanics, prioritize OTI and avoid using non-invasive ventilation and high-flow nasal cannula.

#### Postprocedural Recommendations

- At the end of the procedure, direct the patient to the specific zone for confirmed COVID-19 or for patients waiting for SARS-CoV-2 RT-PCR;
- Wait for transportation inside the procedure room to prevent contamination of the post-anesthesia recovery room.

**Postprocedural Cleaning**

- Remove PPE inside the procedure room;
- Perform final cleaning of the room as usual.

## 7. Electrophysiological Study

**Table:** 

**Classification: Invasive contact of airways**
**Risk: Extremely high**

### a. Elective Procedures

The general recommendations for reopening elective programs apply to the cath lab invasive procedures. Patient’s informed consent should be obtained after clarification of the risks and benefits of undergoing the procedure during the COVID-19 pandemic, and different patients’ flows should be observed for elective and urgency/emergency care.

The most urgent tests/interventions (Table 9) and those with the highest prognostic impact should be prioritized to reduce the risk of death and prevent clinical decompensations.

- Elective procedures in symptomatic patients or in those with a recent positive RT-PCR test, even though asymptomatic, should be postponed.
- Although controversial, the use of RT-PCR testing to diagnose COVID-19 in asymptomatic patients admitted for elective electrophysiological study can be considered adequate to reduce the risk of in-hospital transmission (Figure 3).

### b. Urgency and Emergency Procedures

In the presence of imminent risk of hemodynamic decompensation or death (Table 10), patients with COVID-19 or unknown infectious status may be admitted for emergency interventions. In these cases, the safety recommendations for the healthcare team apply concomitantly.

**Table 10:** Classification of the electrophysiological study procedures during the COVID-19 pandemic^19^

**Elective healthcare environment**
Electrophysiological study or AF, flutter or nodal ablation in stable outpatients
Electrophysiological study to assess stable tachyarrhythmias
Cardioversion of stable arrhythmias with tolerable symptoms
Left atrial appendage closure in patients on oral anticoagulation
Tilt-table testing
**Semi-elective healthcare environment**
Ablation of drug-refractory and recurrent ventricular tachycardia
Supraventricular tachycardia determining multiple visits to the emergency unit
AF, flutter or nodal ablation in patients with recurring symptoms
**Urgency and emergency healthcare environment**
Ablation of ventricular tachycardia due to drug-refractory electrical storm
Wolf-Parkinson-White syndrome or pre-excitation with syncope or cardiovascular arrest
AF, flutter or nodal ablation in patients with significant hemodynamic impairment and drug refractoriness or cardioversion resistance

AF: atrial fibrillation.

The pre-procedural and procedural recommendations are similar to those described for the cath lab.

## 8. Special Considerations on Heart Transplant Patients

COVID-19 has specific repercussions for patients involved in heart transplant, such as donors and recipients, in the waiting list, and after the transplant. As already known, these patients are at increased risk for both SARS-CoV-2 infection and progression to severe disease because of comorbidities, their constant contact with healthcare units and professionals, in addition to immunosuppression. However, these patients are known for their high adherence to medical recommendations.^20^ Thus, targeted prevention and treatment strategies are necessary.^21^

Regarding the donor, non-infected individuals should be chosen, having in mind that many can be asymptomatic/pre-symptomatic/oligosymptomatic carriers and that the current tests have significant limitations. Thus:

- SARS-CoV-2 RT-PCR testing should be performed as soon as organ donation is consented.
- Whenever available, perform chest CT to rule out radiographic findings of suspected infection.
- If the RT-PCR test results positive, the organs should not be used for transplantation.

Regarding in-patients, both in the waiting list and posttransplant management:

- They should be kept in ‘non COVID-19’ units, being cared for by a dedicated multiprofessional team with no contact with positive cases of SARS-CoV-2 infection.
- In-person outside visits should be limited and not recommended. The communication routine with family should be organized.

Regarding the ambulatory posttransplant management, the following is recommended:

- Limit in-person visits for stable and asymptomatic patients.
- Encourage telemonitoring of the serum levels of immunosuppressants;
- Delay endomyocardial biopsy in stable patients.

Heart transplant recipients, due to their sustained immunosuppression, may present typical (respiratory) and atypical (gastrointestinal) manifestations of SARS-CoV-2 infection. In such cases, the following is recommended:

- Consider reducing the calcineurin inhibitor dose (cyclosporine or tacrolimus).
- Suspend temporarily antiproliferative drugs (mycophenolate or azathioprine).

## 9. Guidance to Patients with Risk Factors for SARS-CoV-2 Infection and its Clinical Repercussions

Clinical cardiologists play an essential role in the care of patients with SARS-CoV-2 infection, among whom there is a high prevalence of risk factors associated with more severe clinical repercussions. Thus, the proper control of risk factors is essential for continuing the treatment, for the new decision-making, and for guidance on reducing the infection spread.

### Patients at Increased risk for Severe SARS-CoV-2 Infection

Table 6 shows the risk factors for the severe COVID-19 forms. These patients should be advised on their condition and the need for continuing their treatment:

- They should neither change nor suspend their medications without talking to their physicians.
- They should maintain a minimum medication supply to one month.
- They should maintain their vaccines updated according to the immunization calendar (especially against influenza and pneumococcal disease).
- They should remain physically active and maintain a healthy lifestyle, as a measure to reduce the risk of COVID-19 complications.^22^ The practice of physical exercises according to the recommended safety measures to prevent COVID-19 contamination should be encouraged.
- Fighting against smoking is strongly recommended, even among lower-risk populations, such as young individuals, because of the increased likelihood of complications.^23^
- Never postpone the search for urgent care in case of any warning sign, such as chest pain, dyspnea, changes in speech, gait and/or localized muscular strength, or other condition requiring immediate attention.

These recommendations are aimed at strengthening adherence to treatment and control of cardiovascular risk factors (especially obesity and smoking), a strategy that may add additional value to the reduction in the risk of complications from COVID-19.^24^^,^^25^

In addition, patients should be advised on maintaining the measures to reduce the risk for spread, such as:

- To limit physical proximity interactions with other people, whenever possible.
- To take every precaution when interacting with other people:
  ◦ observe the minimum 2.0 m physical distancing.
  ◦ wear a cloth face mask.
- To promote hand hygiene with water and soap or alcohol gel.
- To contact their physician in case of fever, diarrhea or respiratory symptoms.
- In case of suspected COVID-19, contraindicate self-medication and instruct about its risks.
- To avoid activities in which protective measures cannot be taken, such as situations in which social distancing cannot be maintained (gatherings in closed environments, events).
- To avoid getting close to other people who are not taking protective measures.

### Post-discharge Care of Cardiac Patients and their Return to the Workplace after SARS-CoV-2 Infection

Studies indicate that up to 20% of the patients have cardiovascular complications, such as arrhythmias, acute coronary syndrome, and myocardial injury during their hospitalization from COVID-19.^21^

These patients have a more severe clinical presentation of COVID-19 and mortality three times higher. In addition, they can face several obstacles in their household setting after discharge. The need for rehabilitation should be emphasized for those with post-discharge functional limitations.

- Physical: several patients will be discharged requiring care because of their respiratory limitations or care in wound/pressure areas. In addition, interventions will be needed to recover both the muscle mass and functional capacity of those with neuromyopathy of the critically-ill patient.
- Psychological and neuropsychological: because of their disease and treatment, recovering patients may develop persistent psychological adversities or even cognitive impairment.
- Socioeconomic: the patients’ needs and socioeconomic circumstances have been commonly affected by the COVID-19 pandemic. In addition, the potential impact of the changes during isolation should be considered.

Therefore, patients with severe cardiovascular manifestations of COVID-19 should be reassessed in the first week after hospital discharge regarding cardiovascular symptoms, adherence to medication, and clarification of doubts and difficulties to readapt to routine activities, considering the procedures of rehabilitation described below. In addition, the minimum time to return to work activities should follow the existing guidelines and consider post-discharge functionality and the minimum isolation necessary.

### Cardiovascular Rehabilitation

Scientifically established as an important intervention in secondary prevention, cardiovascular rehabilitation is one of the measures with class IA indication by the SBC in several care contexts (coronary artery disease, postoperative period of cardiac surgery, and heart failure), being particularly important after hospital discharge from acute conditions, such as COVID-19.

Currently, when social distancing and mobility restriction measures are required, the implementation of effective models that combine in-person care and remote telemonitoring is urgent. Worldwide, different means of communication have been used in the virtual rehabilitation process (telephone/mobile, apps for smartphone, e-mail, text message, Internet pages, videoconferences). However, the individual risk-benefit assessment of remote healthcare is essential, such as lower intensity in intensive physical training, less social support, remote training standards still being elaborated, and safety concerns with patients at higher risk. The adaptation of cardiopulmonary rehabilitation settings during the COVID-19 pandemic is detailed in Table 11.

**Table 11:** Recommendations for the adaptation of cardiopulmonary rehabilitation centers for the COVID-19 pandemic

Apply the Screening Questionnaire of Symptoms and Exposures (Appendix A) and cancel in-person care in case of a positive answer to any question
The patient and the team must wear surgical masks during the entire length of stay in the rehabilitation center
Maintain the minimum 2.0 m distance, whenever possible, during the use of the rehabilitation equipment (cycle ergometer, treadmill)
Organize individual sessions or reduce the number of patients per session to the minimum possible
Provide systematic disinfection of the material used before and after each activity
Use shorter programs, concentrating effort in the major components of each patient
Replace, whenever possible, in-person sessions with remote assessment and monitoring, instructing patients according to the equipment and using the most suitable means of communication (telephone, text messages, e-mail, videoconference, dedicated platforms and apps)
Promote special strategies for immunocompromised patients, such as those undergoing heart transplantation
Suspend community activities that do not respect the social distancing measures

## 10. Safety of Patients and Healthcare Professionals During the COVID-19 Pandemic

Protecting patients and healthcare professionals at all levels should be the major objective in resuming work activities during the COVID-19 pandemic. Studies have shown the high contamination rate among healthcare professionals, in addition to that group’s high potential to spread COVID-19, as super transmitters at both the workplace and the community.

Hand hygiene and the proper use of PPE are essential to minimize the contamination risks for healthcare workers by SARS-CoV-2. It is paramount that all healthcare personnel receive training on the proper use of PPE, paying special attention to putting on and removing PPE, which should be standardized to reduce the risk of contamination. All personnel should be trained and show the ability to correctly and safely put on, use, remove, and dispose PPE.^3^^,^^26^

Guidance on the specific PPE use should be based on the biological risk the professionals are exposed to during their activities and follow the recommendations:

- Regulation by certifying agencies and Anvisa.
- Proper use, cleaning or periodical disposal, according to technical recommendations.
- Inspection, repair, and replacement according to the manufacturer’s instructions.

It is worth emphasizing that, in addition to the team’s physical safety, legal, psychological, economic, and information safety should be considered.

### a. Physical Safety

To ensure the physical integrity of healthcare professionals who return to their activities is one of the major goals of implementing safety measures. In addition to the continuous training of the whole team, the daily application of the Screening Questionnaire of Symptoms and Exposures to all professionals and the tracing of contacts of confirmed cases, as well as the PPE provision and its rational and systematic use, are essential.

#### Selection of the Team Upon Return to Work

Services will reopen gradually, thus it is important to start with healthcare professionals at lower risk and to consider adjusting the risk to the workplace the professional is assigned to.

Evidence suggests that younger professionals without risk factors be prioritized to ‘close-contact’ and ‘invasive contact of airways’ environments, where the occupational risk for SARS-CoV-2 infection is higher. On the other hand, older or middle-aged professionals with risk factors should be assigned to provide remote healthcare and/or maintain strict physical distancing. The other cases should follow Table 12.

**Table 12:** Classification of the priorities to consider for the healthcare personnel return

	Young, no risk factor	Middle-aged or young with risk factor*	Elderly or middle-aged with risk factor*
Invasive contact of airways	1	2	3
Close contact	1	2	3
Moderate contact	1	2	2
No contact / Remote	1	1	1

Priority 1 – Return immediately Priority 2 – Return after all priority 1 healthcare professionals Priority 3 – Do not return, unless extremely necessary

* The risk factors are listed in Table 4

#### Measures for Transmission Control and Isolation of Suspected Cases

Healthcare professionals who present suggestive symptoms of SARS-CoV-2 infection must be immediately excluded from the workplace. Then, their contacts in the 4 days preceding symptom onset should be traced. Once identified, these contacts should be monitored, instructed to quarantine for 10 days from the last contact, and, when available, undergo nasopharyngeal swab collection for RT-PCR testing.

#### Criteria for Healthcare Personnel Returning to Work after COVID-19

##### • Suspected cases

The healthcare professional with suspected COVID-19 (negative RT-PCR or not performed) can return to work after meeting both criteria:

1. At least 3 days (72 hours) have passed since clinical recovery, defined as:

- Resolution of fever without the use of fever-reducing medication.
- Improvement in respiratory symptoms (cough, shortness of breath) AND

2. At least 10 days have passed since symptoms first appeared.

##### • Confirmed cases

◦ *Symptomatic cases*

The healthcare professional with confirmed COVID-19 (positive RT-PCR) can return to work after meeting both criteria:

1. At least 3 days (72 hours) have passed since clinical recovery, defined as:

- Resolution of fever without the use of fever-reducing medication.
- Improvement in respiratory symptoms (cough, shortness of breath) AND

2. At least 10 days have passed since symptoms first appeared.

◦ *Asymptomatic cases*

The lack of symptoms prevents these individuals from being assessed based on the phase of disease. Thus, if there is confirmation by a positive RT-PCR test in an asymptomatic patient, the criterion for discontinuing quarantine is:

➢ At least 10 days have passed since the date of their first positive RT-PCR test.

For asymptomatic patients confirmed by use of serological tests, there is no clear guidance on their return to work after a positive test. However, considering the workplace safety, the safest strategy should be observed. Thus, we suggest:

➢ For reagent IgM or IgA serology or reagent IgG/IgM – return 10 days after the test.
➢ For reagent IgG serology – no physical distancing is required.

An infection control expert should be consulted to advise on the return to work of individuals who might remain infectious longer than 10 days (immunocompromised conditions).

#### Medical Residency and Specialization

Interns are an important part of the cardiology programs and services in Brazil. During the COVID-19 pandemic, many of them have been transferred from their imaging internship to the clinical healthcare of patients with COVID-19 at hospitals and intensive care units.

Upon return to their previous activities, beginners should be assigned to sectors where their inexperience does not increase the team’s exposure. Education needs to be reviewed, in the search for new learning methods, including learning approaches based on videoconferences and remote training.

### b. Legal Safety

Hospitalized patients should sign the informed consent, which should preferably state their awareness about the procedure being performed during the COVID-19 pandemic, with risks inherent in the procedure and the exceptional time. In addition, the daily application of the Screening Questionnaire of Symptoms and Exposures to healthcare professionals and patients should be documented and stored, in case of future need, as should be the written documents about the precautionary measures against transmission used by the healthcare team.

#### Healthcare Professionals in Hazardous Settings

As previously mentioned in the section *Selection of the team upon return to work*, some professionals will not resume work immediately. In such cases of absence from work (Tables 6 and 12) or during their symptomatic period, isolation and rehabilitation due to SARS-CoV-2 infection, a supportive network between experts is necessary for the possible immediate referral of patients under their care.

### c. Psychological Safety

As the COVID-19 pandemic continues, strategies to psychologically support healthcare professionals, mainly those more susceptible to emotional distress, are necessary. The psychological support can include counseling and the development of supportive systems among coworkers. The following are part of the plan:^27^^,^^28^

- Monitor constantly the team’s wellbeing, especially when prolonged shifts are required or when they are relocated to unknown areas.
- Facilitate access to mental health and psychosocial support services.
- Maintain active search for professionals with psychological impairment and in burnout situations.
- Demand periodic feedback from collaborators.
- Provide updated accurate information to all collaborators.
- Rotate workers from higher physical/emotional stress to lower stress areas and functions.

### d. Economic Safety

Currently, the increasing costs with the healthcare to patients with COVID-19, compounded by the reduced income, constitute a financial burden to health organizations both in the public and private sectors. The steering committee must keep the financial planning constantly updated, negotiating public transfers in the Brazilian Unified Health System setting, which is even more overloaded in this COVID-19 pandemic, and insuring according to the revenue forecast in the supplementary health setting. Emphasis should be given to the increase in costs associated with the environmental adjustments, PPE use, and reduction in the capacity of care delivery to ensure patients’ physical distancing during their stay in healthcare services.

### e. Information Safety

The fight against fake news and the provision of a proper health literature to inform patients are missions of the cardiologist and a multidisciplinary team. The SBC has issued technical reports to guide cardiologists and patients. The World Health Organization and government agencies have shown concern about the “infodemic” (with the spread of fake news) and its impacts on the patients’ physical and psychological health.

The healthcare team has the responsibility to be updated and provide patients with clear and objective information based on safe sources to prevent the spread of incorrect, incomplete, misunderstood, or fake information.

## 11. Appendix A

Screening Questionnaire of Symptoms and Exposures to be applied before elective interventional procedures

**Questionnaire of symptoms:**

**In the last 14 days did you have any of the following symptoms?**

Major symptoms (only 1 is enough to raise suspicion):

□ Fever
□ Cough
□ Shortness of breath
□ Mental confusion
□ Loss of taste or smell

Minor symptoms (at least 2 are required to raise suspicion):

□ Fatigue/tiredness
□ Diarrhea
□ Runny nose
□ Nausea and/or vomiting
□ Sore throat
□ Headache
□ Conjunctivitis
□ Other:_________________________

**Questionnaire of Exposure:**

1. In the last 14 days did you have contact, for more than 15 minutes and closer than 2.0 m, with an individual diagnosed with COVID-19 or identified by the doctor as a case of suspected COVID-19?

□ Yes □ No

2. In the last 14 days were you admitted to any health service?

□ Yes □ No

3. If healthcare professional: Have you contacted patients with suspected or confirmed COVID-19 without wearing PPE?

□ Yes □ No
